# Food as Medicine: Curbing Type-2 Diabetes Prevalence Through Consumption of High Amylose Starchy Foods in Sub-Saharan Africa

**DOI:** 10.34172/apb.025.43630

**Published:** 2025-02-22

**Authors:** Muyiwa S. Adegbaju, Ifeoluwa E. Adegbaju, Memunat A. Issah, Fatimatou Saccoh, Ademola A. Falade, James R. lloyd, Olanrewaju B. Morenikeji

**Affiliations:** ^1^Institute of Plant Biotechnology, Stellenbosch University, Stellenbosch, South Africa; ^2^Department of Biomedical Sciences, Rochester Institute of Technology 153 Lomb Memorial Drive Rochester NY 14623, Rochester, USA; ^3^Department of Nutrition and Dietetics, Federal University of Technology Akure, Akure, Nigeria; ^4^Department of Biochemistry, Faculty of Chemical and Life Sciences, Usmanu Danfodiyo University, Sokoto, Nigeria; ^5^Division of Biological and Health Sciences, University of Pittsburgh at Bradford, Bradford, PA, United States

**Keywords:** Food system, Type-2 diabetes mellitus, Starch, Sub-Saharan Africa

## Abstract

The prevalence of nutrition-related non-communicable diseases like diabetes mellitus (DM) is exponentially increasing across the world. Particularly, type-2 diabetes mellitus (T2DM) is prevalent in sub-Saharan Africa (SSA) than in any other region of the world, with a significant effect on mortality and morbidity. T2DM is a disease known to be associated with elevated glucose levels in the blood, caused by numerous factors including dietary and lifestyle changes. Ensuring an adequate supply of a healthy diet through a transformed food system could be a potential strategy to mitigate T2DM in SSA. In plants, starch is the most common storage carbohydrate, and it is the major glucose-releasing carbohydrate in human diets. The rate of starch digestibility varies and is largely due to the proportion of its two polyglucan components, amylose and amylopectin. Although, no medication has been found to effectively treat T2DM, it could be managed through effective postprandial glycemia control. This article reviews the mechanism for slowing down the rate of starch digestion and absorption in the small intestine through direct alteration of amylose and amylopectin in starch crops. This strategy would ensure the supply of healthy diets for consumption and ultimately help to curb the increasing prevalence of T2DM.

## Introduction

**Figure 1:**
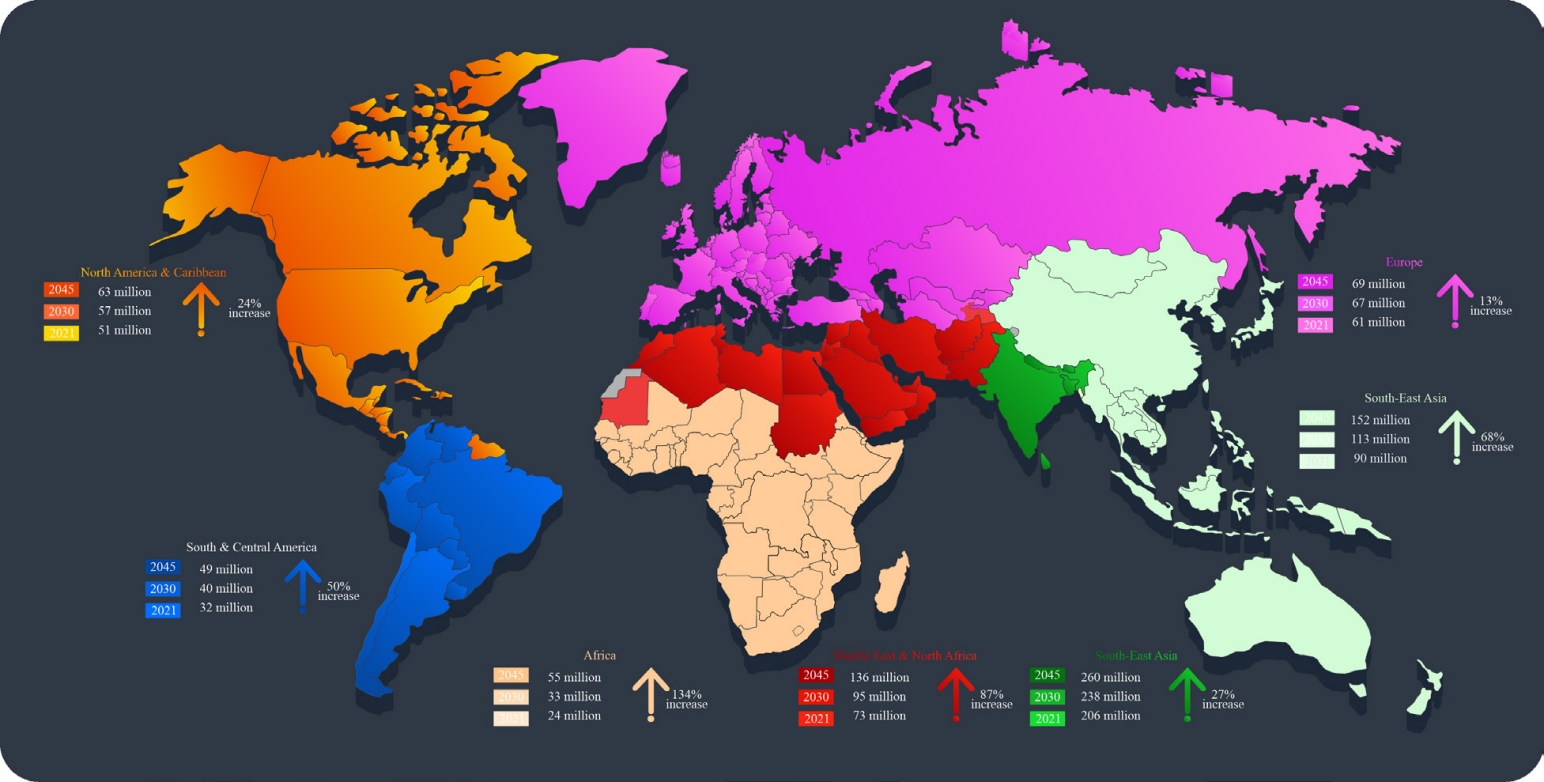
Global diabetes prevalence in 2021. The status combines both T1DM and T2DM data and is based on adults between the ages of 20 and 79. It presented projections for DM in 2030 and 2045, followed by a percentage increase from 2021 to 2045. (Source)^6^

 According to the World Health Organization (WHO), diabetes mellitus (DM) is a chronic metabolic disorder characterized by the pancreas’s inability to produce sufficient insulin or the body’s ineffective use of insulin. Insulin regulates the conversion of starch, sugar, and other foods into glucose, and its proper functioning is crucial for maintaining optimal blood glucose levels.^1^ Diabetes has been on the rise since 2000, and it is now a global public health concern, ranking eighth as the leading cause of death.^2^ The International Diabetes Federation’s 2021 report reveals alarming statistics: approximately 24 million Africans aged 20-79 live with diabetes, and the disease claims nearly half a million lives annually. The top five countries in Africa with the highest diabetes prevalence rates are South Africa, Nigeria, Tanzania, Ethiopia, and the Democratic Republic of Congo. By 2045, projections indicate that Africa will experience the highest rise in diabetes prevalence compared to other regions (Figure 1). This is especially concerning, given that over 70% of diabetes patients in sub-Saharan Africa (SSA) remain undiagnosed.^3-5^ Furthermore, Africa’s already overburdened healthcare systems are grappling with the rising prevalence of diseases such as HIV/AIDS, tuberculosis, and malaria, hence intensifying the diabetes crisis.^2^

 According to Afshin et al,^7^ improving diets could potentially prevent one in every five deaths worldwide, with dietary risks affecting people regardless of sex, age, or socio-demographic development. In Africa, where most countries have low-middle to low socio-demographic index values, this issue is particularly pressing. Insufficient fruit consumption and inadequate whole grain intake contribute significantly to mortality and Disability-adjusted life years in the region. Furthermore, changes in dietary patterns and lifestyle, particularly high carbohydrate consumption, drive the rising incidence of diabetes in Africa.^8,9^ Adopting healthy, low-calorie diets is crucial to curb the prevalence of DM and other diet-related diseases. However, addressing malnutrition in SSA is complicated by several challenges. These include poor diets, limited food variety, low agricultural output and income, insufficient food availability, and climate change impacts.^10,11,12^ These interconnected factors underscore the need for comprehensive solutions that address not only individual dietary choices but also the broader food system and socioeconomic context.

 In 2003, African states approved the Comprehensive Africa Agriculture Development Programme (CAADP) to attain food and nutrition security and eliminate poverty. The CAADP-Malabo declaration, implemented in 2014, further reinforced this, aiming to ensure the availability of healthy foods in African countries.^13^ This requires the transformation and sustainability of Africa’s food systems, a complex network that encompasses food production, transport, processing, and consumption. A sustainable food system will guarantee the provision of a healthy diet to meet current food needs while preserving ecosystems that can ensure a sufficient food supply for future generations with minimal harm to the environment. However, establishing a sustainable food system in Africa will require science, technology, and innovation to accurately estimate the nutritional values of common foods and improve food quality. As a result, this review aims to uncover the link between the prevalence of DM in SSA and changes in its inhabitants’ dietary patterns. We examined and suggested an innovative crop biotechnology approach to improve a major component of common food crops to curb the prevalence of T2DM in the region.

## Evidence and impact of rapid urbanization in sub-Saharan Africa

 SSA is undergoing rapid urbanization, with its global share of urban inhabitants projected to increase from 11.3% in 2010 to 20% in 2050.^14-16^ Governmental policies, infrastructural investments, and technological innovations propel this transition, fostering economic growth but also causing unforeseen effects on dietary practices and health outcomes. The accelerating pace of urbanization and socioeconomic change in SSA has given rise to widespread urban agricultural practices. According to Hemerijckx et al,^17^ urban agriculture in Kampala, Uganda, contributes twice as much to urban food provision as international imports. This suggest that Urban agriculture and food systems can provide access to more nutritious options.^18^ However, these systems may also displace conventional ones, encouraging unhealthy fast-food consumption.^19-22^ Improved transportation networks and food delivery can boost access to options but may lead to sedentary lives.^23-26^ Additionally, technological innovations can enhance the efficiency of food production and delivery, but they may foster nutritional quality compromise while prioritizing convenience and profit.^27,28^

 Urbanization, while offering potential for economic growth and increased income opportunities,^29,30^ has yet to deliver on its promise in SSA.^31^ A substantial slum population in the region is an indication that a lot of urban migrants lack access to better living conditions.^32^ Clear economic disparity has been created because of this and it affects various income groups distinctly, with significant implications for dietary patterns and health outcomes. A 2008 survey in Cape Town, South Africa, exemplified this, showing that 80% of households in low-income regions faced challenges in obtaining adequate food, while 68% suffered from extreme food insecurity.

 Rapid urbanization, urban poverty, and global food inflation, according to Dorosh et al,^33^ appear to have created a perfect storm, leading to reduced access to health diets and food security and a troubling increase in instances of poor diets. This is markedly worrisome, as studies persistently show that unhealthy food choices and physical inactivity caused by sedentary lifestyles are leading risk factors for noncommunicable diseases, including T2DM.^34,35^ Additionally, it seems that factors such as the expansion of urban centers into neighboring rural communities, the ease of communication between urban and rural areas, the ties between urban and rural dwellers, and numerous other factors blur the rural-urban divide on dietary changes. As a result, the prevalence of diabetes in Africa has dramatically increased in both males and females equally, regardless of their geographic location.^36^ Thus, deciphering the complex interplay between urbanization, economic growth, and dietary changes is crucial for addressing food security and nutrition challenges in the region.

## Dietary shifts in Sub-Saharan Africa: Evidence of increasing starch-based diet

 In Africa, urbanization’s impact on food choices deviates from global trends.^30^ Contrary to expectations that rising wealth would increase animal-based product consumption, Africa’s urbanization has fueled significant growth in per capita rice consumption.^37,38^ This shift aligns with prevailing plant-based diets in African homes, characterized by starchy staples and limited animal proteins, fruits, and vegetables.^39^ This reliance on staple foods has profound implications for dietary patterns. In Nigeria, staple foods exceed the recommended 34.0% daily calorie intake, accounting for 52.5% and 66.8% of calories in urban households with highest and lowest incomes, respectively. Similarly, rural households rely heavily on staples, with 60% and 76% of daily calories for wealthiest and poorest homes.^40^ In another study, Chiaka et al^41^ found that Nigerian households across geographical zones derive over 70% of daily calories from cereals and starchy roots. This persistent dominance of carbohydrate-rich diets challenges the notion of urbanization-driven nutritional transition towards diverse, high-protein foods.^42,43^ Consequently, this dietary pattern contributes to rising non-communicable nutrition-related disorders in SSA,^9,44^ underscoring the need for tailored nutritional interventions.

## Diagnosis, economic impact, management of diabetes mellitus

 There are two distinct forms of DM: Type 1 diabetes mellitus (T1DM) and type 2 diabetes mellitus (T2DM), which have long been recognized by the medical community and facilitating targeted research, diagnosis, and management strategies.^45^ T1DM is a complex condition triggered by a combination of genetic and environmental factors, leading to an autoimmune response that ultimately results in a complete deficiency of insulin production. This condition typically emerges during childhood or adolescence. On the other hand, the T2DM condition is characterized by elevated blood sugar levels, insufficient insulin production, and insulin resistance.^46^ These factors impede the body’s capacity to efficiently use the insulin it produces, thereby impairing glucose regulation.^47^ The subtle onset of T2DM can lead to a significant delay in diagnosis, often taking 9-12 years after the disease begins.^48^ Typically, T2DM is diagnosed later in life, often after the age of 40. However, in Africa, where T2DM accounts for a staggering 90% of all diabetes cases,^49^ a concerning trend has emerged: a recent surge in T2DM incidence among children and adolescents, deviating from its traditional association with older adults.^48^

 Diagnosing diabetes in Africa is a complex challenge, hindered by the similarity in symptoms between diabetes and HIV/AIDS, as well as socioeconomic and cultural barriers. These factors often lead to misdiagnosis or delayed diagnosis, further complicated by inadequate healthcare infrastructure, resulting in a staggering 70% of diabetes cases in SSA remaining undiagnosed.^3-5^ For instance, a 2008 report in Nigeria revealed 2.5 million cases of undiagnosed diabetes,^50^ highlighting the severity of underdiagnosis in the region. Limited access to healthcare facilities, inadequate training of healthcare providers, and insufficient screening efforts contribute to this underdiagnosis, ultimately leading to preventable early deaths due to delayed or lack of treatment. As sub-Saharan African populations rapidly urbanize, diabetes incidence and prevalence are likely to increase, exacerbating the issue. Therefore, addressing these deficiencies and strengthening diabetes detection and management in the region is crucial to combat this growing burden.

 The challenges of diagnosing diabetes in Africa are part of a larger global issue, with far-reaching economic implications. A staggering report by Diabetes Research and Clinical Practice in 2019 revealed that diabetes and its complications claimed over four million lives worldwide, with nearly half of these deaths occurring among individuals under 60.^51,52^ This trend is particularly alarming, as it not only poses a growing threat to younger populations, but also has significant economic implications, given that this age group comprises the backbone of any society’s workforce. Delayed diagnosis exacerbates this issue, leading to serious complications like diabetic nephropathy, retinopathy, and erectile dysfunction, which increase healthcare costs, reduce workforce participation, and result in productivity losses. Timely detection and effective management are crucial to mitigate these economic impacts. While pharmaceutical treatments exist, concerns about their long-term safety persist.^53,54^ Therefore, adopting a healthy lifestyle, coupled with timely detection and effective management of T2DM, is vital to reducing premature deaths, improving health outcomes, and increasing life expectancy.^55^ By taking proactive steps, individuals can prevent or delay the onset of complications, ultimately minimizing the economic burden of diabetes.

 The intake of easily digestible carbohydrates, especially starch with specific physicochemical characteristics, can have a direct impact on blood glucose levels, posing significant risks for T2DM. Two key factors that contribute to the development of T2DM are insulin resistance and oxidative stress, which are triggered by repeated fluctuations in postprandial glycemia.^56^ In other words, consuming easily digestible carbohydrates can lead to spikes in blood sugar levels, increasing the likelihood of developing insulin resistance and oxidative stress, both of which are precursors to T2DM.

 Extensive research has investigated factors influencing postprandial glycemia, revealing that both the type and quantity of carbohydrates consumed^57-59^ and interactions between starch and other food components—particularly starch granule characteristics^60-62^—significantly impact blood sugar control following meals. This knowledge underscores the complexity of carbohydrate digestion and absorption, emphasizing the need to consider multiple factors when evaluating the glycemic impact of foods. Effective diabetes management hinges on regulating postprandial glycemia, which helps minimize complications.^63^ This often requires lifestyle modifications, including dietary changes.^64^ Given that starch is the primary carbohydrate in most consumed foods in SSA, it is crucial to refocus research efforts on developing strategies to make dietary starch safer and healthier for consumption, ultimately reducing the risk of diabetes-related complications in this region.

## Nutritional importance of starch

 Glucose is the body’s essential fuel, powering the functioning of all tissues and organs. Notably, the brain consumes a substantial amount of glucose, utilizing around 25% of the body’s basal metabolic energy.^65^ Other vital organs and tissues, including the kidneys, reproductive tissues, and red blood cells, also depend on a steady glucose supply to maintain proper function. Moreover, during pregnancy and lactation, glucose demand surges to support the energy-intensive processes of fetal growth and milk production, highlighting its critical role in these life stages.^66,67^

**Figure 2:**
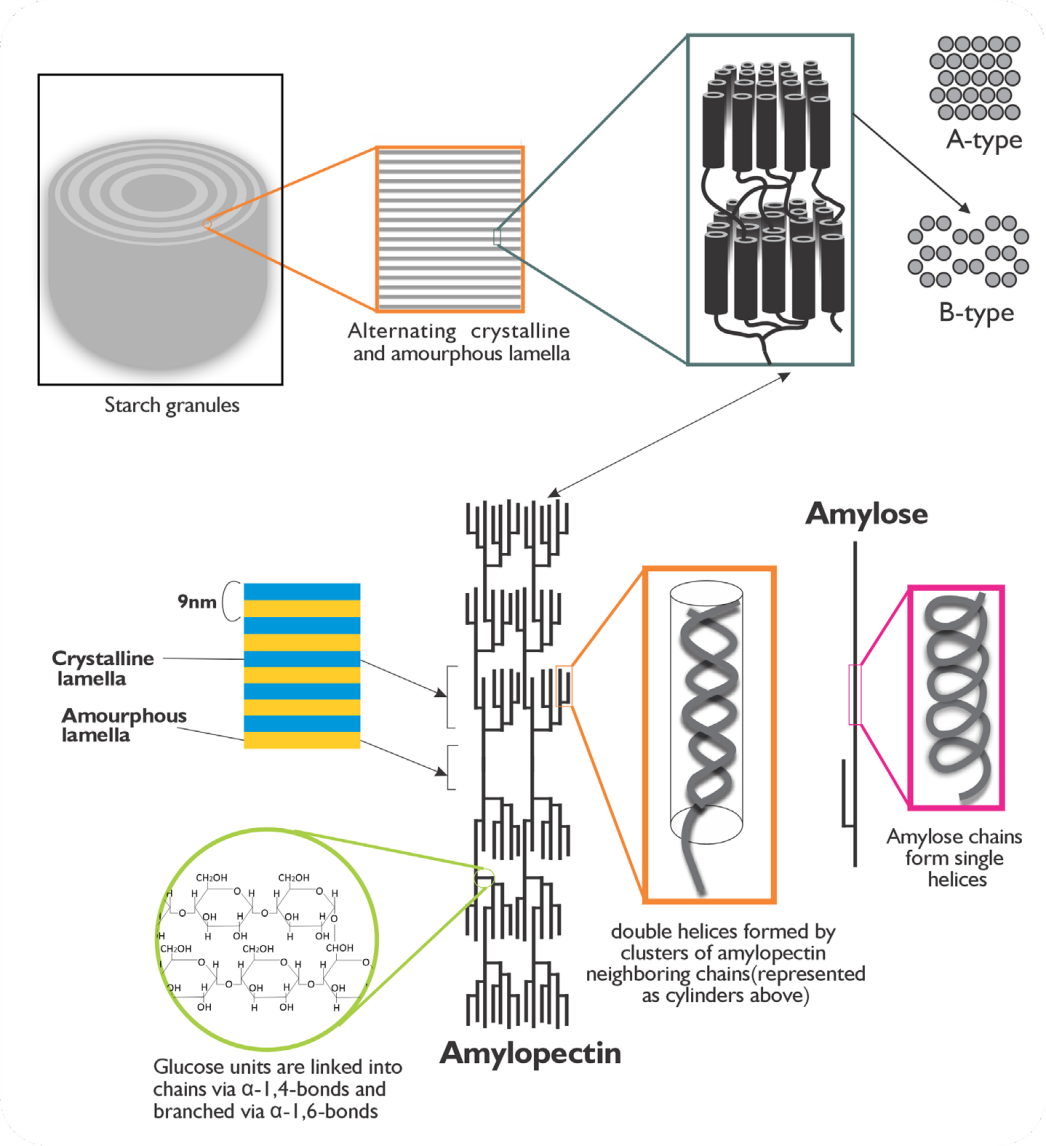
Structural composition of starch granules. Schematic representation of amylopectin and amylose structure. Amylose has molecular weight of ~10^5^-10^6^ kDa and its chains is made up of about 1000 or more glucosyl residues in length. Amylopectin consists of alternating crystalline―composed of glucan chain double helices created from contiguous chains within a cluster―and amorphous lamellae―the branch-point zone linking two clusters―which are repeated at distance of 9 nm down the molecule’s axis. The double-helices crystallize into two types of polymorph patterns A or B and the third type C-polymorphic pattern which is admixture of A and B patterns. Image was re-drawn from Zeeman et al.^69^

 Dietary carbohydrates primarily meet the body’s fundamental glucose needs by releasing glucose in the form of starch, primarily found in the storage organs of common food crops such as rice, wheat, corn, potato, and cassava (endosperm, roots, and tubers). These starch-rich crops, listed in Table 1, dominate global agricultural land use, with a substantial portion of arable land dedicated to their cultivation.^69^ As a result, they play a vital role in meeting the world’s glucose requirements. Depending on the plant source, angiosperms store starch in plastids as distinct granules with varying shapes and sizes.^70^ These granules comprise two primary polymers, amylose, and amylopectin, present in varying proportions (Table 2). The molecular structures of amylose and amylopectin differ significantly. Amylose is a predominantly linear polymer (Figure 2) comprising α-1,4-linked D-glucose monomers, with occasional branching at α-1,6 linkages.^71,72^ In contrast, amylopectin is a highly branched molecule composed of α-1,4-linked D-glucose units, with approximately 5% of its linkages being α-1,6 branches.^73-86^ The unique properties of these two polymers significantly affect starch functionality and performance during thermal processing and culinary applications.

**Table 1:** Composition of major food crops (in % by fresh weight)

**Crop**	**Starch (%)**	**Protein (%)**	**Lipid (%)**	**Ash (%)**	**Moisture (%)**
Rice	73.8	6.8	2.7	1.2	15.5
Maize	70.6	8.6	5.0	1.3	14.5
Cassava	26	1	0.3	0.2	66
Wheat	72.2	10.6	3.1	1.6	12.5
Sweet Potato	31.5	1.2	0.2	1.0	66.1
Potato	17.6	1.6	0.1	0.9	79.8
Banana	22.5	1.1	0.2	0.8	75.4

Note: the data represent a standard table of food composition from Japan in 2010.^68^

**Table 2:** Proportion of amylose and amylopectin in starch granules of various starch sources

**Starch source**	**Amylose (%)**	**Amylopectin (%)**	**DPn**^a^	**CLn**^b^	**DPn**^c^
Rice	8–37	63 – 92	1015	300	n.a
Maize	20–36	64 – 80	895	323	2 000 000
Cassava	17	83	4000	340	2 000 000
Wheat	17–29	71 – 83	1275	203	2 000 000
Sweet Potato	19–20	80 – 81	3280	335	n.a
Potato	18–23	77 – 82	5630	595	2 000 000
Sorghum	21–35	65 – 79	n.a^f^	n.a	n.a
Barley	11–26	74 – 89	1450	413	n.a

Note: Data represents summary from the following sources.^73-86^ The amylose content of starches from plants varies not only according to botanic source but also based on cultivars, here the values for amylose content of certain crops were derived from examining 399 (maize), 74 (rice), 493 (potato), 284 (sorghum), 167 (Wheat) and 61 (barley) cultivars. ^a^ average degree of polymerisation (glucosyl units) of amylose; ^b^ average chain length of amylose (glucosyl units); ^c^ average degree of polymerization of amylopectin (glucosyl units); ^f^ not available.

## Starch digestion in the human

**Figure 3:**
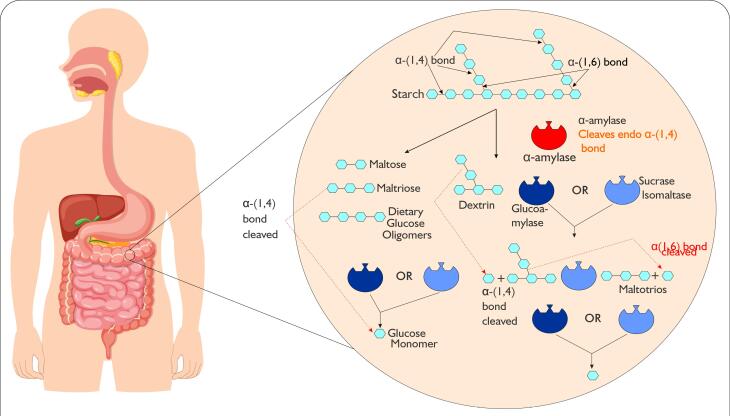
Schematic diagram of starch breakdown in the small intestine

 The breakdown of starch begins in the mouth, where salivary alpha-amylase initiates the process through its hydrolytic activity.^87^ However, this enzyme is inactivated in the acidic environment of the stomach. The digestion of starch then continues in the small intestine, where pancreatic alpha-amylase takes over, further hydrolyzing the starch molecules into less smaller products such as alpha-limit-dextrins, maltose and maltotriose. These products are further hydrolyzed into glucose by enzyme complexes sucrase-isomaltase and maltase-glucoamylase located in the brush border membrane (Figure 3).^88^ The small intestine rapidly absorbs the glucose and use it as energy for various cellular processes.^89^

 Starches exhibit varying digestibility rates, categorizing them into three primary fractions: Rapidly digestible starch (RDS), slowly digestible starch (SDS), and resistant starch (RS).^90^ Consuming RDS-rich foods triggers rapid blood glucose and insulin spikes, increasing the risk of developing T2DM and cardiovascular diseases.^91,92^ Conversely, SDS undergoes gradual digestion in the small intestine, resulting in a slow and sustained glucose release into the bloodstream.^92,93^ Meanwhile, RS remains undigested in the small intestine and is fermented in the colon.^94,95^

## Types of resistant starch

 Based on indigestible mechanisms and structural features, RS is generally categorized into five types.

- Resistant starch 1 (RS_1_) is physically unreachable to digestive enzymes because it is locked in the food matrix, which consists of cell wall materials and proteins within partially milled or whole grains and tubers. For instance, the protein matrix locks away the RS_1_ in pasta, making it the wheat product with the highest RS content.^96^
- RS_2_ are native starch granules with B- or C-type polymorphic patterns (Figure 2). They are hard to digest because they are packed together tightly, making it hard for digestive enzymes to get to them. Native and uncooked starch granules that can be eaten raw, like potatoes, unripe bananas, and plantains, are in this category; however, the RS content decreases significantly after cooking while the RDS increases concomitantly.^97,98^ This is another example of RS_2_-type starches, which are increasingly important as preferred dietary starches because their long-chain double-helical crystallites make them stable even after cooking^99^ and subsequently cooling, leading to retrogradation.^100^
- RS_3_ retrogrades more rapidly, forming extra-resistant crystallites. Both amylose and amylopectin fractions reassociate during retrogradation to create closely packed double helices, stabilized by hydrogen bonds. The structure formed by retrogradation makes them more resistant to hydrolytic enzymes.^84^ Amylose and amylopectin, composed of long glucan chains,^101-103^ are preferable molecules for the formation of RS_3_. On the other hand, short-chain amylopectin takes up to several days to retrograde.
- RS_4_ refers to native starches that undergo chemical modification or re-polymerization. They include starches that have been esterified, etherified, or cross-linked with chemicals.^104^ These chemical changes slow down the breakdown of starch because they form steric hindrances at the sites where enzymes work.^105,106^
- RS_5_, as described by many reports, is an amylose-lipid complex—a single helical structure formed between amylose and lipids.^107-110^ However, RS_5_ also includes resistant maltodextrin that results from sequentially administering pyroconversion and enzymatic hydrolysis to native starch.

## Resistant starch and type 2 diabetes management: health benefits and mechanisms

 Individuals with T2DM have impaired insulin sensitivity, leading to elevated glucose levels. Consequently, starch intake can potentially worsen glucose control and contribute to disease progression due to its rapid digestion, causing blood glucose spikes measured by its glycemic index (GI). Foods with high GI disrupt postprandial glucose homeostasis, leading to recurrent hyperglycemia, insulin resistance, and increased T2DM risk. In contrast, foods rich in RS have a low GI, lower energy density, and offer numerous health benefits. Notably, high-amylose starchy foods slow down starch digestion and absorption in the small intestine. Amylose’s unique structure forms a compact, resistant helix, limiting digestive enzyme access. Specifically, α-amylase hydrolyzes amylopectin more efficiently than amylose, resulting in a slower glucose release from amylose-rich starches. This slower digestion and absorption lower postprandial glucose spikes, improving insulin function and potentially reducing T2DM incidence.^94,95,111,112^ International organizations, including the FAO, WHO, and European Association for the Study of Diabetes, recommend categorizing foods by their GI to manage blood glucose levels.^113,114^ However, GI is only one piece of the puzzle, as other factors significantly influence glycemic response. The type of nutrients present, the rate of gastric emptying, insulin release, and incretin activity―particularly glucagon-like peptide-1 (GLP-1) and glucose-dependent insulinotropic polypeptide (GIP)―all play critical roles.^115,116^ Notably, the American Diabetes Association (ADA) advises against relying solely on GI for managing T2DM, instead emphasizing the importance of total dietary carbohydrate content and available insulin.^117^ According to the ADA, these factors have a more profound impact on glycemic response than the type or source of carbohydrates. Research on carbohydrate sources and their impact on glucose, insulin, and incretin responses has yielded promising findings. For instance, a study in healthy men found that consuming high-amylose rice alleviated postprandial hyperglycemia and hyperinsulinemia without affecting gastric emptying rate or GLP-1 secretion.^118^ Similarly, Maki et al^119^ demonstrated that high-amylose maize consumption improved insulin sensitivity in overweight and obese males. These studies suggest that high-amylose starchy foods are superior carbohydrates and functional foods for diabetes management.

 Given the growing consumption of starchy foods in SSA, it is crucial to prioritize research that enhances and enriches common starchy foods with low-digestible starch, thereby improving their nutritional value. This targeted approach can potentially mitigate the rise of diabetes and related disorders in the region.

## Modification of starch functionality through a biotechnological approach

 The functionality of starch has been effectively modified through physical, enzymatic, and chemical methods. However, there is a growing consumer demand for healthy and high-quality foods produced using innovative, environmentally friendly, and clean production systems. As a result, there is significant interest in directly modifying starch functionality within crops due to its universality, economic and dietary importance, simple chemical structure, and well-understood biochemical pathways of starch biosynthesis.^120^ Biotechnology has enabled the manipulation of key enzymes involved in starch biosynthesis, leading to the development of new starches with high amylose content in commonly grown crops. Notably,^121^ found a link between high amylose content in starch and RS. Therefore, this discussion will begin with an overview of starch biosynthesis in plants, followed by an examination of biotechnological advancements aimed at increasing amylose content.

## Starch synthesis in plants

**Figure 4:**
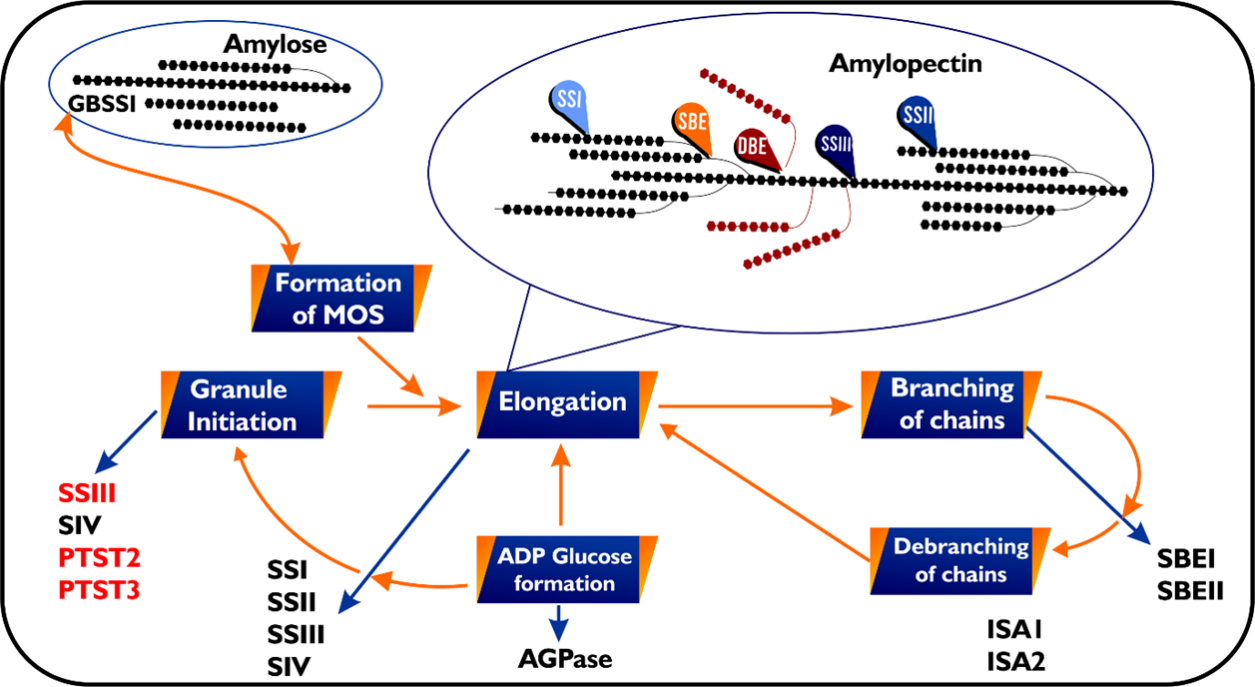
A schematic representation of enzyme-mediated reactions involved in amylose and amylopectin biosynthesis in plants. The diagram depicts the interlink of non-linear reactions of different starch biosynthetic enzymes. The process of starch synthesis starts with the synthesis of ADP glucose used as glucosyl donor. Amylose is synthesised by the activity of a special starch synthase GBSS,^123^ Under different condition GBSS can switch from processive mode of action (addition of more than one glucose unit per substrate encounter thereby facilitating the synthesis of long linear chains^124^) to distributive mode of action (Elongation of glucan chains by one glucose unit per encounter),^125^ which determines its affinity for malto-oligosaccharide (MOS).^126^ Through distributive mode of action three isoforms of SS elongate amylopectin chains. Branching of amylopectin is created by SBEs from existing chains and misplaced branches are removed by two isoforms of isoamylase-type DBEs. Granule initiation occurs through the activities of two isoforms of SSs and two proteins Protein targeting starch (PTST) 2 and 3. The Orange arrows indicate the various steps involved in starch synthesis while blue arrows indicate isoforms of various enzyme classes involved in each step.

 ADP-glucose pyrophosphorylase starts the process of starch biosynthesis in plants by making ADP-glucose. This sugar then acts as a glucosyl donor in the biosynthesis process.^122^ Several starch synthases (SSs) add a glucose ADP-glucose unit to an acceptor chain at the nonreducing end (Figure 4), which makes the glucan chain longer. Starch branching enzymes (SBEs), an enzyme group, catalyze amylopectin synthesis by cleaving a linear chain and conveying the released fragment to a C6 hydroxyl group of the same or adjacent chain. The activity of SBEs makes more substrates, i.e., non-reducing ends of an acceptor chain, available for the SSs to elongate. Amylopectin’s cluster structure forms with the help of debranching enzymes (DBEs), a different type of hydrolytic enzyme. This class of enzyme reduces excess branches by hydrolyzing branched linkages.

 Angiosperms duplicated the SS, SBE, and DBE genes, resulting in multiple forms of the enzymes they encode. Although studies have shown that most of these isoforms perform specific roles during starch biosynthesis, they often exhibit overlapping functions. Granule-bound starch synthase (GBSS), a distinct type of SS, solely catalyzes the reaction that synthesizes amylose.^123^ Many plants commonly display five isoforms of soluble starch synthases (SSI, SSII, SSIII, SSIV, and SSV), and researchers have elucidated their specific functions in both monocots and dicots. Researchers have implicated three of the isoforms in the elongation of amylopectin chains, each with a preferred chain length they act upon: SSI catalyzes the elongation of short chains with 6-7 degrees of polymerization (DP) to 8-12 DP; SSII forms intermediate chains of 13-25 DP; and SSIII mainly catalyzes the formation of both intermediate and very long chains.^127-135^ Together, the three SS isoforms control structural formation and determine the cluster size of amylopectin.^120^ On the other hand, SSIV has been implicated in starch granule initiation.^136,137^ The report by Abt et al^138^ also demonstrated that SSV plays a role in starch granular initiation in the chloroplast. Even though it is a noncanonical isoform, it shares structural similarities with SSIV.

 The diversity of starch-branching enzyme (SBE) isoforms varies across plant species. Cereals possess three SBE isoforms (SBEI, SBEIIa, and SBEIIb), whereas potatoes have only two. According to,^139,140^ SBEs were classified into the A-family and B-family. For example, pea SBEI, maize SBEII, and potato SBEII belong to the A-family, while rice SBEI, maize SBEI, pea SBEII, and potato SBEI belong to the B-family.^139,141-145^ Research reveals that SBE isoforms differ in their amylopectin branch formation capabilities, with B-family members transferring longer chains than A-family members.^146-150^ In potatoes, SBEI (B-family) exhibits a preference for long linear substrates like amylose, whereas SBEII (A-family) favors branched substrates like amylopectin.^149^

 SBEs play a big role in figuring out how amylopectin branches and how its chain lengths are distributed, which is what makes up its cluster structure.^151,152^ DBEs come in two types: isoamylase (ISA) and limit dextrinase (LDA or pullulanase). Three isoforms of isoamylase have been identified, in contrast to a single form of limit dextrinase.^153-155^ ISA1 and ISA2 are essential for starch biosynthesis, while ISA3 and LDA primarily contribute to starch degradation.^154,156-158^ Isoamylase’s role is critical, as it enhances amylopectin crystallization by ‘trimming’ misplaced branches, thereby promoting structural order.^120,159^

## Innovative approaches for gene modification to alter starch biosynthesis

 Modern biotechnology tools can now manipulate certain starch biosynthetic enzymes to produce high-amylose starch in various crops more quickly than traditional plant breeding techniques, thanks to significant advancements in molecular genetics over the past two decades and conventional modifications in plant breeding. In the following sections, we will expand upon various manipulations of some of the starch biosynthetic enzymes that led to a significant increase in amylose content (Table 3). We will also describe the effects of such manipulations on starch yield.

**Table 3:** Crops modified for increase amylose content through various genetic modification methods

**Plant species**	**Cultivar(s)/Parent(s)**	**Inactivated gene/s**	**Starch amount versus WT**	**Percentage increase in amylose content**	**Modification** **approach**	**Reference**
Pea	*rugosus* (*r*) pea	*SBEI*	50%^p^ lesser	100%	Insertion of a transposon-like element into the coding Sequence (Natural)	^160^
Rice	Japonica cv. Kinmaze (*EM10*)	*SBEIIb*	55%^q^ lesser	66%	Chemical mutagenesis	^161-163^
	Japonica cv. Ilpumbyeo		Not available	45%	Chemical mutagenesis	^164^
	Indica cv. Te-qing	*SBEI/ SBEIIb*	Not available	138%	RNAi	^165^
	Japonica cv. Nipponbare	*SBEIIb*	Unaltered	110%	RNAi	^101^
	Japonica cv. Kitaake	*SBEIIb*	Unaltered	66.7%	CRISPR-Cas9	^166^
	Japonica cv. Nipponbare	*SBEIIb*	26%^r^ lesser	40%	CRISPR-Cas9	^167,168^
	Japonica cv. Nipponbare and Kinmaze	*SS3a/ BEIIb*	22%^s^ lesser	45%	Cross between *SS3a* and *BEIIb* mutants	^169^
Maize	Amylose extender maize	*SBEIIb*	30%^s^ less starch	50%	Natural mutation	^170,171^
	High amlose donor (USA) and HKI 1344, HKI 1378, HKI 1348-6-2 (India)	*SBEIIb or ae1*	~22%^s^	49.3%	Cross between *ae1 *donor and hybrid parents	^172^
	Inbred line Chang 7-2	*SBEIIa&SBEIIb*	10.5%^p^ lesser	92.3%	RNAi	^173^
	Inbred line Tie7922	*SBEIIa*	Unaltered	87.2%	RNAi	^174^
Wheat		*SBEIIa*-A^-1^, *SBEIIa*-B^-1^&*SBEIIa*-D^-1^	~43%^r^ lesser	210%	Heavy-ion beam irradiation	^175^
	Kronos	*SBEIIa*-A &*SBEIIa*-B	n.d	61%	Chemical mutagenesis	^176^
	Mountrail/PI 330546 Mountrail/IG 86304 (*SSIIa*-A^-1^)	*SSIIa*-B	49%^r^ less starch	41%	Chemical mutagenesis	^177^
	Cadenza	*SSIIIa*-A/*SSIIIa*-B/*SSIIIa*-D	41%^r^ lesser	35%	TILLING	^178^
	Kronos& Express	DW (*SBEIIa*-A &*SBEIIa*-B) and BW (*SBEIIa*-A, *SBEIIa*-B &*SBEIIa*-D)	DW (12%^p^) and BW (11.7%^p^)	DW (94%) and BW (142%)	TILLING	^179^
	Svevo	*SBEa*-A^-1^&*SBEa*-B^-1^	31%^r^ less starch	96%	TILLING	^180^
	Cadenza	*SBEa*-B^-1^&D^-1^	10%^r^	17% - 20%	Chemical mutagenesis	^181^
	n.a	*SBEIIa*	16.5%^s^ less starch	192%	RNAi	^182^
	Svevo	*SBEIIa*	25%^q^ less	206%	RNAi	^183^
Barley	Himalaya	*SSIIa /Sex6*	176%^q^ less starch	184%	Chemical mutagenesis	^184^
	High amylose Glacier	*SSiiia /amo1*	10%^r^ less starch	40%		^185^
	Golden Promise	S*BEIIa*&*SBEIIb*	32%^r^ less starch	213%	RNAi	^186^
	Golden Promise	*SBEI*, *SBEIIa*&*SBEIIb*	12%^q^ less starch	No amylopectin detected	RNAi	^187^
Cassava	TMS60444	*SBEII*	n.a	127.3%	RNAi	^188^
Potato	Desiree	SBEI &SBEII	50%^q^ less starch	161%	Antisense Technology	^189^
	Prevalent, Producent and Dinamo	*SBEI*&*SBEII*	Less starch (no value)	139%, 136% and 142%	Antisense Technology	^190^
	Kuras and Dinamo	*SBEI*&*SBEII*	n.a	295%	RNAi	^191^
	Desiree	All *SBEI*&*SBEII* alleles	unaltered	No amylopectin detected	CRISPR-Cas9	^192,193^

Note: Cv; Cultivar, SBE; starch branching enzyme; DW; durum wheat, BW; bread wheat; A; A genome, B; B genome, C; genome, -1; null, n.a; not available. p; dry weight, q; grain fresh weight, r; Whole meal/grain flour, s; endosperm.

## Increasing amylose content in starch by manipulation of starch biosynthesis

###  Modification of SSs activities

 Modifications in the activities of three starch synthases (SSs) responsible for amylopectin biosynthesis have been shown to lead to varying elevations in amylose content and alterations in the chain length of amylopectin branches. Rice, specifically *Oryza sativa*, is categorized into two main cultivated subspecies: japonica-type and indica-type, as distinguished by.^194^ Foods made from cultivars of the two subspecies have different textures because their amylose content and amylopectin structure are very different.^195,196^ These textural differences have been attributed to the polymorphic variations in the *gbssi* and *ssiia* genes, which are specific to each group. A single nucleotide polymorphism identified in the japonica cultivar, compared to the indica cultivar, is associated with reduced activities of the GBSSI and SSII enzymes.^195-197^ A nucleotide polymorphism within intron 1 of the GBSSI transcript in Japonica rice leads to aberrant splicing, resulting in an unusually long transcript.^198,199^ This genetic variation directly affects amylose content in Japonica endosperm starch, reducing it by approximately 5-10% compared to Indica rice.^199,200^ Furthermore, a reduction in SSIIa activity in japonica rice caused the amylopectin chain to increase from DP6 to 12 and a decrease in those with DP13 to 24,^201,202^ resulting in a reduction in starch gelatinization peak temperature when compared with the Indica cultivar.^195^ Recent research by Crofts et al^203^ showed that incorporating active SSIIa and/or high-expressing GBSS1 alleles from indica rice into japonica rice ss mutants increases gelatinization temperature. This is attributed to altered amylopectin chain length distribution and elevated amylose content. Similarly, *Oryza glaberrima*, Africa’s native cultivated rice species, exhibits higher amylose content and gelatinization temperature compared to *Oryza sativa*.^204^ Notably, Wambugu et al^205^ identified a non-synonymous SNP in the GBSS1 gene, potentially influencing enzyme activity and contributing to differences in starch properties.

 According to Fujita et al^128^ where mutant rice plants deficient in SSI was manufactured, it exhibited a modest increase in both total and apparent amylose content in their starch. Notably, mutant rice plants lacking SSIIIa showed a more pronounced effect, with a 1.5-fold increase in amylose content A subsequent study by Fujita et al^206^ found that rice plants with simultaneous suppression of both Starch Synthase I (SSI) and Starch Synthase IIIa (SSIIIa) exhibited a slightly higher starch amylose content compared to plants with only SSIIIa suppression, indicating a cumulative effect of the double suppression on amylose content. On the contrary, Japonica rice mutants lacking *ssiia* showed no increase in amylose content, although there was a decrease in amylopectin chain length.^201^

 The barley mutant, which produces endosperm starch with an elevated amylose content (45%), was first identified by Merritt^207^ and attributed the phenotype to a gene named *amo1*.^208^ Later, Li et al^185^ identified SSIIIa as the potential gene whose mutation caused high amylose in the amo1 mutant. However, more research by Li et al^185^ showed that the phenotypic effect seen in the amo1 mutant might not be due to the complete loss of SSIIIa activity, but rather to the negative regulation of other genes involved in starch biosynthesis. On the other hand, a mutation in *ssiia* conferred a high amylose phenotype on the endosperm starch of the *Sex6* barley mutant.^184^ While this Ssii/Sex6 mutant’s amylopectin had shorter chains, the endosperm’s lipid level went up a lot.^209^ This may have helped the formation of a complex between lipids and amylose (RS_5_), which is what led to the high level of RS found in foods made from this barley mutant.^210^

 In a similar vein, Yamamori et al^211^ documented a wheat line called “starch granule protein-1 null” that exhibits a lack of expression of all three *SSIIa* genes. Further study revealed that this is caused by mutations involving a deletion in the exon sequences of the A and D genomes, as well as an insertion in the B genome.^212^ The null wheat line exhibits a similar composition of amylose and amylopectin with shorter chains in its endosperm starch, resembling the Sex6 barley mutant phenotype.^211,213^ The presence of premature stop codons in the *ssiiia* of genomes A, B, and D (triple mutants) and AB, AD, and BD (double mutants) of hexaploid bread wheat resulted in elevated amylose levels as determined by the SEC method. Nevertheless, it was shown that only the triple mutant exhibited a substantial elevation in amylose levels as determined by both the SEC and iodine techniques.^178^ The RS content in whole meal flour and purified starch of the triple mutants were more than twice as high as that of the control, according to Sestili et al.^183^ This implies that altering SS activity in certain crops can lead to an increased quantity of amylose in the store starch.

 A common feature in grains of mutants lacking SS activity is shrunkeness.^178,184^ Previous studies have shown that deficiency in the activity of important starch biosynthetic enzymes often results in a reduced amount of starch in the embryo, accompanied by an increased sucrose level, which causes the shrunkenness of mature seeds.^160,214^ The grains of the barley amo1 mutant line^185^ are full and plump, even though there is a small decrease in the amount of starch compared to the wild type. On the other hand, the barley s*ex6* mutant line and wheat *SSiiia* triple mutants have smaller grains.

 Further research has elucidated the role of SS in starch structure formation across various crops, including potatoes and peas. Interestingly, concurrent inhibition of SSI and SSII in potatoes did not yield an expected increase in amylose content within the tuber starch. This is likely because these isoforms have overlapping activities in this crop, as stated by Kossmann et al.^215^ Furthermore, the sucrose level did not increase, even though the starch content in the tuber remained unchanged.^215^ Therefore, it is reasonable to suggest that *ss* mutants, particularly in cereal crops, may produce less amylopectin, resulting in a higher proportion of amylose, even though the actual amount of amylose remains unchanged. According to Craig et al,^216^ a mutation in the *ssII* gene caused the development of long-chain amylopectin in the pea rug-5 mutants first generated by Wang et al.^217^

###  Modification of SBE activities

 High amylose starch has been detected in mutant or transgenic plants through genetic modification of the SBE gene. The presence of high amylose starch, reaching up to 50%, was initially discovered in monocot plants, specifically in *amylose extender* (*ae*) maize, and a dicot plant known as *rugosus* (*r*) pea was found to contain even higher levels of amylose starch, ranging from 65% to 70%. The two natural mutants were designated based on the phenotype they displayed.^170,218^ Later, Horan and Heider^73^ verified that the observed characteristics were a result of a genetic mutation in the starch branching enzyme of *amylose extender*. Additionally, White^219^ identified *rugosus* (*r*) as the specific locus affected by the mutation. The specific enzymes responsible for causing this phenotype were eventually identified as SBEIIb in maize extender and SBEI in the (*r*) mutant pea.^160,220,221^ Therefore, the inherent reduction in starch branching enzyme activity in both *ae* maize and *r* pea is directly associated with the elevated amylose content of the mutants. Several food products, like white bread, incorporate high-amylose starch from maize, which is readily available in the market. However, incorporating RS directly into the diet through wholegrain or wholemeal offers additional nutritional benefits compared to using it as a supplementary ingredient from purified starch. We describe various efforts to manipulate SBE in other notable cereals, roots, and tuber crops to increase the amylose content.

 Researchers have manipulated each of the SBEs in rice; for instance, mutant rice deficient in SBEI did not alter the amylose content of the endosperm, despite a minor increase in the short chains of amylopectin and a decrease in the long chains.^166,222^ The first rice cultivar to have RS is ‘EM10’, also known as super-hard rice, a mutant lacking SBEIIb’s activity. The endosperm starch of this rice variety has a significant amount of amylose, as well as amylopectin with a longer molecular chain.^162^ These phenotypes have been further confirmed in two cultivars of japonica rice where *sbeiib* was mutated^166,168^ and in trangenic Japonica rice where *SBEIIb* was repressed.^101^ Additionally, Zhu et al^165^ found that simultaneous repression of both SBEI and SBEIIb increased the amount of amylose in the endosperm starch of Indica rice. Although Zhu et al^165^ did not determine the amount of starch, it likely decreased.

 This is because starch accounts for approximately 90% of the dry weight of rice kernels, and the 38% reduction in kernel weight reported in there study could likely be attributed to a decrease in starch accumulation in the endosperm. The study by, Baysal et al^168^ which showed a 26% reduction in the starch amount of dry seed, also showed a reduction in dry seed and dehulled grain weights.

 The possibility of increasing the amylose in wheat was demonstrated in the transgenic wheat repressed in *sbeiia*.^183,223^ In this study, a decrease in *SBEIIb* expression occurred concomitantly with that of *SBEIIa* which was originally targeted and it was established that this was not as a result of cross-silencing of sbeiib alongside sbeiia but due to concerted activities of the two enzymes which are necessary for amylopectin synthesis.^223^ However, sole repression *SBEIIb* did not show any significant alteration in the starch composition or structure.^223^ This trend was also analogous in barley as only the repression of *SBEIIa* by RNAi – not true for *SBEIIb* repression – caused an increase of amylose to about 50%.^186^ Transgenic barley that is repressed in the three *SBE* genes produced starch granules with only amylose.^187^

 Transgenic wheat with suppressed *SBEIIa* demonstrated the potential to enhance the amylose content in wheat.^183,223^ The study observed a decrease in *SBEIIb* expression in conjunction with the original target, *SBEIIa*. Researchers concluded that the coordinated actions of these two important enzymes for amylopectin synthesis, not the simultaneous repression of *SBEIIb* and *SBEIIa*, caused this drop.^223^ Nevertheless, the exclusive inhibition of *SBEIIb* did not result in any notable modification in the content or structure of starch.^223^ Only the inhibition of *SBEIIa* using RNAi led to an approximately 50% increase in amylose content in barley, while the repression of *SBEIIb* did not have the same effect.^186^ Genetically modified barley suppressing its three SBE genes produces solely amylose-containing starch granules.^187^

 In potato repression of SBEI activity leads to no increase in amylose and minor alterations in starch structure,^144,192,193^ but decreases in SBEII activity lead to a minor increase in the amylose content of the tuber starch and an increase in small starch granules.^145^ Transgenic repression of activities of both SBEI and SBEII leads to large increases in amylose in potatoes^191,190^ and in mutants potato where all the four alleles of both *Stsbei* and *Stsbeii* are mutated, only amylose accumulates.^192,193^ Likewise amylose content increased in storage starch of cassava in which *SBEII *wasrepressed.^188^

 The modification of SBEs is a critical factor in increasing amylose content. By reducing amylopectin biosynthesis while maintaining a constant amylose proportion, and forming long amylose-like chains on amylopectin, SBE modification leads to a relative increase in amylose proportion. This is achieved through decreased branching frequency on amylopectin, resulting in the formation of long, amylose-like chains. Furthermore, interactions between SBE isoforms and other starch biosynthetic enzymes may also impact amylopectin branching, contributing to the biosynthesis of starch.^189,224,225^ Ultimately, SBE modification plays a pivotal role in regulating amylose content in various crops.

## Benefits and limitations of high amylose starchy food

 By harnessing the power of genetic engineering to optimize the starch biosynthesis pathway, scientists have achieved a substantial increase in amylose content across various crops. This innovation has cleared the path for the development of new, amylose-rich cultivars, poised to make a significant impact on the food industry and enhance the nutritional value of staple crops. The introduction of high-amylose rice cultivars in SSA could have far-reaching benefits for public health. Previous studies have suggested that regular rice consumption may increase the risk of developing DM. Research, however, has shown that high-amylose rice products, such as cookies made from high-amylose rice flour, can help regulate blood sugar levels.^226^ Additionally, studies in both normal and diabetic rats have demonstrated the positive effects of high-amylose rice grains on glucose metabolism.^165^ Also, tests on humans have shown that eating high-resistant starch rice lowers the rise in blood sugar and insulin levels after a meal.^227^ This shows that high-amylose rice may be able to lower the risk of diabetes and improve health outcomes in the SSA region as a whole. Human clinical trials have validated the health benefits of high-amylose barley grains.^197^ In a groundbreaking development, Australian scientists have engineered a genetically modified barley variant, dubbed BARLEYmax, by eliminating the *ss2a* and *ss3a* genes.^185^ This innovative variant boasts an exceptionally high amylose content, making it an ideal ingredient for various food products, including breakfast cereals, flatbreads, cereal bars, porridges, and more. BARLEYmax has paved the way for the creation of nutritious and functional food options, harnessing the advantages of high-amylose barley to support public health.

 Corrado et al^228^ performed an extensive study examining the effects of high-amylose wheat flour bread on starch digestibility and glycemic response. Their findings revealed that, compared to conventional bread, starch breakdown was reduced by 20% in vitro, while in vivo glycemic response decreased by 15%. These results are similar to the general trend of better glycemic control, but they are a little different from those of Belobrajdic et al,^62^ who found a stronger effect, with a 39% drop in post-meal glycemic response and a 24% drop in insulinemic response after eating high-amylose wheat flour bread. The underlying mechanism driving the reduced postprandial glycemic response in both studies likely stem from the decreased amount and availability of carbohydrates in high-amylose wheat bread. Moreover, the compact structure of high-amylose starch may restrict starch swelling and gelatinization, thereby slowing the rate and extent of digestion.^229,230^

 Furthermore, an earlier study by Van Hung et al^231^ showed that blending regular wheat flour with high-amylose flour in a 1:1 ratio produced bread that was not only acceptable in terms of quality but also retained a significant amount of RS. Corroborating this, Corrado et al^228^ found that incorporating high-amylose flour into bread did not compromise its texture or appearance, suggesting that this could be a viable strategy for creating functional bread products with enhanced nutritional profiles.

 Recently De Arcangelis et al^232^ examined the impact of replacing durum wheat semolina with high-amylose bread wheat flour in pasta production. Their study involved substituting semolina at varying levels (30%, 50%, and 70%) and found significantly increased RS content in cooked pasta products. Notably, high-amylose flour substitution slowed starch digestion rates across all samples, suggesting potential benefits for glycemic control and digestive health. The 70% high-amylose semolina-type flour composition exhibited optimal cooking and nutritional properties. Rice varieties engineered to lack SBEIIb activity, either through mutation or genetic modification, have shown significant nutritional enhancements. However, these improved rice lines share a common characteristic―opaque rice grains―unlike the typically translucent appearance of conventional rice.^101,162,165,167^ Although the flour from these nutritionally enhanced rice varieties could be valuable in various food products, such as wheat and rice bread and noodles,^233^ the opaque grain appearance may detract from their appeal. This aesthetic change could impact consumer acceptance and marketability, presenting a challenge to the adoption of these improved rice lines despite their potential nutritional benefits.

 A common trait among mutant or transgenic rice varieties is that elevated amylose content often leads to a decrease in endosperm starch.^102,167,168^ However, a notable exception was observed when indica’s *GBSSI* and *SSIIa* genes were introgressed into Japonica rice lacking SBEIIb activity through the crossing. This resulted in a mutant rice line that not only maintained a similar endosperm starch content to regular indica rice but also exhibited increased amylose content. Although, this mutant rice had approximately 10% less endosperm starch compared to conventional Japonica rice.^234,235^ This finding suggests that careful genetic manipulation can mitigate the typical trade-off between amylose content and starch quantity in rice.

 Researchers have also reported a reduction in the storage starch of potatoes and wheat lacking SBE activity, resulting in an increased amylose content.^183,190^ A previous review by Lloyd and Kossmann^236^ examined the biotechnological modification of plants to increase starch content in storage organs. The review also provides suggestions on how to enhance starch yield through biotechnological methods, which may apply to plants that lack SBE activities and have reduced storage starch. Moreover, by scaling up the cultivation of high-amylose rice varieties to harness Africa’s vast arable land resources―which account for 60% of the world’s total―the issue of low starch content be effectively addressed. The continent’s untapped agricultural potential offers a prime opportunity to expand production, driven by the compelling health benefits associated with these cultivars. By leveraging this land availability, the global supply of nutritious, high-amylose crops can be increased, ultimately contributing to improved public health outcomes and economic growth through export to other regions.

## Sub-Saharan Africa’s Regulatory Status on GMOs and Genome-Edited Products

 The scarcity of natural high-amylose crops has spurred innovation in biotechnology, enabling the development of genetically modified (GM) or genome-edited crop varieties with improved amylose content. This breakthrough has significant implications for enhancing nutritional quality and addressing public health concerns. However, the adoption of GM crops and genome editing technologies in SSA is shaped by diverse regulatory frameworks and policy implications. Country-specific regulatory approaches to GM crops and gene editing have resulted in a heterogeneous landscape of governance. GM crops face significant barriers in Africa due to concerns regarding exogenous DNA, consumer skepticism, and high approval costs. Stringent regulatory scrutiny and debates about unintended environmental consequences have further constrained their adoption.^237^ In contrast, genome editing techniques, which may not introduce foreign DNA, have sparked optimism about their potential exemption from GM regulations.^238,239^

 This distinction has led four countries to establish guidelines for regulating genome-edited products, with most exempting products lacking foreign DNA from regulations with south Africa being the only exemption. Notably, South Africa and Kenya have enacted labeling requirements for GMO products, although Kenya exempted certain genome-edited products. The African Union’s Agenda 2063 endorses gene editing as a revolutionary breeding tool with vast agricultural potential, promising more flexible and efficient crop development when exempt from GM regulations.^240^ Numerous African nations with less strict regulations concerning certain GEd products may also gain mutual advantages through a continental framework such as the African Continental Free Trade Area (AfCFTA) declaration. This will promote cross-border trading of high-amylose starchy foods when developed and facilitate the distribution of such items across numerous countries in SSA.

## Conclusion

 Data on the food system in SSA suggests a shift in food demand and preference due to the rise of the middle class and rapid urbanization. It is therefore plausible that the increase in T2DM prevalence in SSA can be associated with dietary changes that favour the consumption of calorically dense and easily digestible starchy foods. Here we discussed a promising way of delaying the onset or management of T2DM, which is to increase the RS component in common starch foods to facilitate the slow release of glucose when ingested. We presented literature-based evidence demonstrating the effective application of modern agricultural biotechnology methods to enhance the RS content in common starchy crops. Some countries around the world have already effectively utilized this innovative technology, leading to the commercialization of various products with high-resistant starch. As African governments continue to make efforts to transform their food systems, they must take advantage of these biotechnology methods for developing starchy crops with high-resistant starch, making them healthier for consumption and ultimately helping to curb the increasing prevalence of T2DM.

## Competing Interests

 Authors declare we have no competing financial or personal interest

## Ethical Approval

 Not applicable.

## References

[1] Aljerf L, Alhaffar I (2017). Salivary distinctiveness and modifications in males with diabetes and Behçet’s disease. Biochem Res Int.

[2] World Health Organization (WHO). The Top 10 Causes of Death. WHO; 2021. Available from: http://www.who.int/mediacentre/factsheets/fs310/en/. Accessed April 7, 2025.

[3] Mbanya JC, Motala AA, Sobngwi E, Assah FK, Enoru ST (2010). Diabetes in sub-Saharan Africa. Lancet.

[4] Fasanmade OA, Dagogo-Jack S (2015). Diabetes care in Nigeria. Ann Glob Health.

[5] Pastakia SD, Pekny CR, Manyara SM, Fischer L (2017). Diabetes in sub-Saharan Africa - from policy to practice to progress: targeting the existing gaps for future care for diabetes. Diabetes MetabSyndrObes.

[6] International Diabetes Federation (IDF). Diabetes Facts and Figures. Available from: https://idf.org/about-diabetes/diabetes-facts-figures/.

[7] Afshin A, Sur PJ, Fay KA, Cornaby L, Ferrara G, Salama JS (2019). Health effects of dietary risks in 195 countries, 1990-2017: a systematic analysis for the Global Burden of Disease Study 2017. Lancet.

[8] Godman B, Basu D, Pillay Y, Mwita JC, Rwegerera GM, Anand Paramadhas BD (2020). Review of ongoing activities and challenges to improve the care of patients with type 2 diabetes across Africa and the implications for the future. Front Pharmacol.

[9] Popkin BM (2006). Global nutrition dynamics: the world is shifting rapidly toward a diet linked with noncommunicable diseases. Am J Clin Nutr.

[10] Hasegawa T, Fujimori S, Takahashi K, Masui T (2015). Scenarios for the risk of hunger in the twenty-first century using shared socioeconomic pathways. Environ Res Lett.

[11] GNR. Global Nutrition Report: Action on Equity to End Malnutrition (Development Initiatives, 2020). 2020. Available from: https://globalnutritionreport.org/reports/2020-global-nutrition-report/. Accessed December 2, 2022.

[12] Aljerf L, Aljerf NJ (2023). Food products quality and nutrition in relation to public Balancing health and disease. Prog Nutr.

[13] Africa Common Position on Food Systems Food Security Leadership Dialogue (ACPOFS). Regional Submission to UN Food Systems Summit 2021. Available from: https://nepad-aws.assyst-uc.com/publication/africa-common-position-food-systems.

[14] Echendu AJ, Okafor PC (2021). Smart city technology: a potential solution to Africa’s growing population and rapid urbanization?. Dev Stud Res.

[15] Saghir J, Santoro J. Urbanization in sub-Saharan Africa. In: Meeting Challenges by Bridging Stakeholders. Washington, DC: Center for Strategic & International Studies; 2018. p. 1-7.

[16] FAO, IFAD, UNICEF, WFP, WHO. The State of Food Security and Nutrition in the World 2023: Urbanization, Agrifood Systems Transformation and Healthy Diets Across the Rural–Urban Continuum. Rome: FAO; 2023.

[17] Hemerijckx LM, Nakyagaba GN, Sseviiri H, Janusz K, Eichinger M, Lwasa S (2023). Mapping the consumer foodshed of the Kampala city region shows the importance of urban agriculture. NPJ Urban Sustain.

[18] Zezza A, Tasciotti L (2010). Urban agriculture, poverty, and food security: empirical evidence from a sample of developing countries. Food Policy.

[19] Boué C, Lopez-Ridaura S, Rodríguez Sánchez LM, Hellin J, Fuentes-Ponce M (2018). Local dynamics of native maize value chains in a peri-urban zone in Mexico: the case of San Juan Atzacualoya in the state of Mexico. J Rural Stud.

[20] Moustier P, Renting H. Urban agriculture and short chain food marketing in developing countries. In: de Zeeuw H, Drechsel P, eds. Cities and Agriculture: Developing Resilient Urban Food Systems. New York: Routledge; 2015. p. 121-38. doi: 10.4324/9781315716312.

[21] Emperaire L, Eloy L (2015). Amerindian agriculture in an urbanising Amazonia (Rio Negro, Brazil). Bull Lat Am Res.

[22] Follmann A, Willkomm M, Dannenberg P (2021). As the city grows, what do farmers do? A systematic review of urban and peri-urban agriculture under rapid urban growth across the Global South. Landsc Urban Plan.

[23] Wachira LJ. Lifestyle transition towards sedentary behavior among children and youth in sub-Saharan Africa: a narrative review. In: Marques A, Gouveia ÉR, eds. Sedentary Behaviour - A Contemporary View. IntechOpen; 2021. doi: 10.5772/intechopen.95840.

[24] Katzmarzyk PT, Mason C (2009). The physical activity transition. J Phys Act Health.

[25] Musau EG, Pisa NM, Masoumi HE (2023). Association between transport-related physical activity and wellness in sub-Saharan Africa: a systematic literature review. Transp Res InterdiscipPerspect.

[26] Muthuri SK, Wachira LJ, Leblanc AG, Francis CE, Sampson M, Onywera VO (2014). Temporal trends and correlates of physical activity, sedentary behaviour, and physical fitness among school-aged children in sub-Saharan Africa: a systematic review. Int J Environ Res Public Health.

[27] Domguia EN, Hymette LF, Nzomo JT, Ngassam SB, Donfouet O (2023). How important is ICT for reducing undernourishment in Africa?. Telematics and Informatics Reports.

[28] Srour B, Fezeu LK, Kesse-Guyot E, Allès B, Debras C, Druesne-Pecollo N (2020). Ultraprocessed food consumption and risk of type 2 diabetes among participants of the NutriNet-Santé prospective cohort. JAMA Intern Med.

[29] de Bruin SP, Dengerink J. The Impact of Urbanisation on Food Systems in West and East Africa: Opportunities to Improve Rural Livelihoods. The Hague: PBL Netherlands Environmental Assessment Agency; 2020.

[30] De Vos K, Janssens C, Jacobs L, Campforts B, Boere E, Kozicka M (2024). African food system and biodiversity mainly affected by urbanization via dietary shifts. Nat Sustain.

[31] Thurlow J, Dorosh P, Davis B. Demographic change, agriculture, and rural poverty. In: Campanhola C, Pandey S, eds. Sustainable Food and Agriculture. Academic Press; 2019. p. 31-53. doi: 10.1016/b978-0-12-812134-4.00003-0.

[32] de Brauw A, Mueller V, Lee HL (2014). The role of rural–urban migration in the structural transformation of sub-Saharan Africa. World Dev.

[33] Dorosh PA, Thurlow J. Agricultural growth, urbanization, and poverty reduction. In: Otsuka K, Fan S, eds. Agricultural Development: New Perspectives in a Changing World. Washington, DC: International Food Policy Research Institute (IFPRI); 2021. p. 285-320. doi: 10.2499/9780896293830_09.

[34] GBD 2019 Diseases and Injuries Collaborators (2020). Global burden of 369 diseases and injuries in 204 countries and territories, 1990-2019: a systematic analysis for the Global Burden of Disease Study 2019. Lancet.

[35] GBD 2015 Risk Factors Collaborators (2016). Global, regional, and national comparative risk assessment of 79 behavioural, environmental and occupational, and metabolic risks or clusters of risks, 1990-2015: a systematic analysis for the Global Burden of Disease Study 2015. Lancet.

[36] Mbanya JC, Assah FK, Saji J, Atanga EN (2014). Obesity and type 2 diabetes in sub-Sahara Africa. Curr Diab Rep.

[37] Herrero M, Havlík P, McIntire J, Palazzo A, Valin H. African Livestock Futures: Realizing the Potential of Livestock for Food Security, Poverty Reduction and the Environment in Sub-Saharan Africa. Geneva: United Nations System Influenza Coordination (UNSIC); 2014. doi: 10.13140/2.1.1176.7681.

[38] Seck PA, Touré AA, Coulibaly JY, Diagne A, Wopereis MC. Africa’s rice economy before and after the 2008 rice crisis. In: Realizing Africa’s Rice Promise. Wallingford UK: CABI; 2013. p. 24-34. doi: 10.1079/9781845938123.0024.

[39] Arimond M, Ruel MT (2004). Dietary diversity is associated with child nutritional status: evidence from 11 demographic and health surveys. J Nutr.

[40] Ecker O, Comstock A, Babatunde R, Andam K. Poor Dietary Quality is Nigeria’s Key Nutrition Problem. Department of Agricultural, Food, and Resource Economics, Michigan State University; 2020.

[41] Chiaka JC, Zhen L, Xiao Y (2022). Changing food consumption patterns and land requirements for food in the six geopolitical zones in Nigeria. Foods.

[42] Seto KC, Ramankutty N (2016). Hidden linkages between urbanization and food systems. Science.

[43] Wanyama R, Gödecke T, Chege CG, Qaim M (2019). How important are supermarkets for the diets of the urban poor in Africa?. Food Secur.

[44] Battersby J, Watson V (2018). Addressing food security in African cities. Nat Sustain.

[45] Olokoba AB, Obateru OA, Olokoba LB (2012). Type 2 diabetes mellitus: a review of current trends. Oman Med J.

[46] Aljerf L, Mashlah A (2017). Characterization and validation of candidate reference methods for the determination of calcium and magnesium in biological fluids. Microchem J.

[47] Dessie G, Mulugeta H, Amare D, Negesse A, Wagnew F, Getaneh T (2020). A systematic analysis on prevalence and sub-regional distribution of undiagnosed diabetes mellitus among adults in African countries. J Diabetes MetabDisord.

[48] Harris MI, Klein R, Welborn TA, Knuiman MW (1992). Onset of NIDDM occurs at least 4-7 yr before clinical diagnosis. Diabetes Care.

[49] Levitt NS (2008). Diabetes in Africa: epidemiology, management and healthcare challenges. Heart.

[50] Whiteney EN, Whiteney E, Rolfes SR. Hypertention. In: Understanding Nutrition. 11th ed. Belmont: Thompson Wadsworth; 2008. p. 632-6.

[51] Saeedi P, Petersohn I, Salpea P, Malanda B, Karuranga S, Unwin N (2019). Global and regional diabetes prevalence estimates for 2019 and projections for 2030 and 2045: results from the International Diabetes Federation Diabetes Atlas, 9th edition. Diabetes Res Clin Pract.

[52] International Diabetes Federation (IDF). Diabetes Atlas. 10th ed. IDF; 2021. Available from: https://diabetesatlas.org/idfawp/resource-files/2021/07/IDF_Atlas_10th_Edition_2021.pdf.

[53] Sharma B, Balomajumder C, Roy P (2008). Hypoglycemic and hypolipidemic effects of flavonoid rich extract from Eugenia jambolana seeds on streptozotocin induced diabetic rats. Food Chem Toxicol.

[54] Niu CS, Chen W, Wu HT, Cheng KC, Wen YJ, Lin KC (2010). Decrease of plasma glucose by allantoin, an active principle of yam (Dioscorea spp), in streptozotocin-induced diabetic rats. J Agric Food Chem.

[55] Global Burden of Metabolic Risk Factors for Chronic Diseases Collaboration. Cardiovascular disease, chronic kidney disease, and diabetes mortality burden of cardiometabolic risk factors from 1980 to 2010: a comparative risk assessment. Lancet Diabetes Endocrinol 2014;2(8):634-47. doi: 10.1016/s2213-8587(14)70102-0. PMC4572741 24842598

[56] Blaak EE, Antoine JM, Benton D, Björck I, Bozzetto L, Brouns F (2012). Impact of postprandial glycaemia on health and prevention of disease. Obes Rev.

[57] Brand-Miller JC, Atkinson FS, Gahler RJ, Kacinik V, Lyon MR, Wood S (2010). Effects of PGX, a novel functional fibre, on acute and delayed postprandial glycaemia. Eur J Clin Nutr.

[58] Lockyer S, Nugent AP (2017). Health effects of resistant starch. Nutr Bull.

[59] Maziarz MP, Preisendanz S, Juma S, Imrhan V, Prasad C, Vijayagopal P (2017). Resistant starch lowers postprandial glucose and leptin in overweight adults consuming a moderate-to-high-fat diet: a randomized-controlled trial. Nutr J.

[60] Hallström E, Sestili F, Lafiandra D, Björck I, Ostman E. A novel wheat variety with elevated content of amylose increases resistant starch formation and may beneficially influence glycaemia in healthy subjects. Food Nutr Res 2011;55. doi: 10.3402/fnr.v55i0.7074. PMC3162347 21876685

[61] Castro-Acosta ML, Lenihan-Geels GN, Corpe CP, Hall WL (2016). Berries and anthocyanins: promising functional food ingredients with postprandial glycaemia-lowering effects. Proc Nutr Soc.

[62] Belobrajdic DP, Regina A, Klingner B, Zajac I, Chapron S, Berbezy P (2019). High-amylose wheat lowers the postprandial glycemic response to bread in healthy adults: a randomized controlled crossover trial. J Nutr.

[63] Parkin CG, Hinnen DA, Tetrick DL (2011). Effective use of structured self-management of blood glucose in type 2 diabetes: lessons from the STeP study. Clin Diabetes.

[64] Ley SH, Hamdy O, Mohan V, Hu FB (2014). Prevention and management of type 2 diabetes: dietary components and nutritional strategies. Lancet.

[65] Fonseca-Azevedo K, Herculano-Houzel S (2012). Metabolic constraint imposes tradeoff between body size and number of brain neurons in human evolution. Proc Natl Acad Sci U S A.

[66] Parrettini S, Caroli A, Torlone E (2020). Nutrition and metabolic adaptations in physiological and complicated pregnancy: focus on obesity and gestational diabetes. Front Endocrinol (Lausanne).

[67] Zhu XZ, Deng ZM, Dai FF, Liu H, Cheng YX (2023). The impact of early pregnancy metabolic disorders on pregnancy outcome and the specific mechanism. Eur J Med Res.

[68] Fujita N. Manipulation of rice starch properties for application. In: Nakamura Y, ed. Starch: Metabolism and Structure. Tokyo: Springer Japan; 2015. p. 335-69. doi: 10.1007/978-4-431-55495-0_10.

[69] Zeeman SC, Kossmann J, Smith AM (2010). Starch: its metabolism, evolution, and biotechnological modification in plants. Annu Rev Plant Biol.

[70] Smith AM, Zeeman SC (2020). Starch: a flexible, adaptable carbon store coupled to plant growth. Annu Rev Plant Biol.

[71] Koroteeva DA, Kiseleva VI, Krivandin AV, Shatalova OV, Błaszczak W, Bertoft E (2007). Structural and thermodynamic properties of rice starches with different genetic background Part 2 Defectiveness of different supramolecular structures in starch granules. Int J Biol Macromol.

[72] Kozlov SS, Blennow A, Krivandin AV, Yuryev VP (2007). Structural and thermodynamic properties of starches extracted from GBSS and GWD suppressed potato lines. Int J Biol Macromol.

[73] Horan FE, Heider MF (1946). A study of sorghum and sorghum starches. Cereal Chem.

[74] Whistler RL, Weatherwax P. Amylose content of Indian corn starches from north, central, and south American corns. Cereal Chem 1948:25:71-5.

[75] Deatherage WL, MacMasters MM, Vineyard ML, Bear RP (1954). A note on starch of high amylose content from corn with high starch content. Cereal Chem.

[76] Goering KJ, Eslick RF, Ryan CJ Jr (1957). Some effects of environment and variety on the amylose content of barley. Cereal Chem.

[77] Juliano BO, Albano EL, Cagampang GB (1964). Variability in protein content, amylose content and alkali digestibility of rice varieties in Asia. Philippine Agriculturist.

[78] Medcalf DG, Gilles KA (2021). Wheat starches I Comparison of physicochemical properties. Cereal Chem.

[79] Reyes AC, Albano EL, Briones VP, Juliano BO (1965). Genetic variation, varietal differences in physicochemical properties of rice starch and its fractions. J Agric Food Chem.

[80] Goering KJ, Eslick R, DeHaas BE (1970). Barley starch IV A study of the cooking viscosity curves of twelve barley genotypes. Cereal Chem.

[81] Miller OH, Burns EE (1970). Starch characteristics of selected grain sorghums as related to human foods. J Food Sci.

[82] Ao Z, Jane J-l. Characterization and modeling of the A-and B-granule starches of wheat, triticale, and barley. Carbohydrate polymers. 2007;67(1):46-55.

[83] Rao SN, Juliano BO (1970). Effect of parboiling on some physicochemical properties of rice. J Agric Food Chem.

[84] Kongseree N, Juliano BO (1972). Physicochemical properties of rice grain and starch from lines differing in amylose content and gelatinization temperature. J Agric Food Chem.

[85] Simek J (1974). Amylosegehalt in der starke von kartoffelweltsortiment. ZeszProblPostepowNaukRoln.

[86] Bertoft E (2017). Understanding starch structure: recent progress. Agronomy.

[87] Svihus B, Hervik AK (2016). Digestion and metabolic fates of starch, and its relation to major nutrition‐related health problems: a review. Starch.

[88] Lin AH, Lee BH, Nichols BL, Quezada-Calvillo R, Rose DR, Naim HY (2012). Starch source influences dietary glucose generation at the mucosal α-glucosidase level. J Biol Chem.

[89] Robyt JF. Enzymes and their action on starch. In: BeMiller J, Whistler R, eds. Starch: Chemistry and Technology. 3rd ed. San Diego: Academic Press; 2009. p. 237-92. doi: 10.1016/b978-0-12-746275-2.00007-0.

[90] Huang J, Yang Q, Pu H. Slowly digestible starch. In: Jin Z, ed. Functional Starch and Applications in Food. Singapore: Springer; 2018. p. 27-61. doi: 10.1007/978-981-13-1077-5_2.

[91] Lehmann U, Robin F (2007). Slowly digestible starch–its structure and health implications: a review. Trends Food Sci Technol.

[92] Miao M, Jiang B, Cui SW, Zhang T, Jin Z (2015). Slowly digestible starch--a review. Crit Rev Food Sci Nutr.

[93] Zhang G, Hamaker BR (2009). Slowly digestible starch: concept, mechanism, and proposed extended glycemic index. Crit Rev Food Sci Nutr.

[94] Harbis A, Perdreau S, Vincent-Baudry S, Charbonnier M, Bernard MC, Raccah D (2004). Glycemic and insulinemic meal responses modulate postprandial hepatic and intestinal lipoprotein accumulation in obese, insulin-resistant subjects. Am J Clin Nutr.

[95] Ells LJ, Seal CJ, Kettlitz B, Bal W, Mathers JC (2005). Postprandial glycaemic, lipaemic and haemostatic responses to ingestion of rapidly and slowly digested starches in healthy young women. Br J Nutr.

[96] Gelencsér T, Gál V, Hódsági M, Salgó A (2008). Evaluation of quality and digestibility characteristics of resistant starch-enriched pasta. Food Bioprocess Technol.

[97] Zhang G, Ao Z, Hamaker BR (2006). Slow digestion property of native cereal starches. Biomacromolecules.

[98] Zhang G, Venkatachalam M, Hamaker BR (2006). Structural basis for the slow digestion property of native cereal starches. Biomacromolecules.

[99] Chiu YT, Stewart ML (2013). Effect of variety and cooking method on resistant starch content of white rice and subsequent postprandial glucose response and appetite in humans. Asia Pac J Clin Nutr.

[100] Shevkani K, Singh N, Bajaj R, Kaur A (2017). Wheat starch production, structure, functionality and applications—a review. Int J Food Sci Technol.

[101] Butardo VM, Fitzgerald MA, Bird AR, Gidley MJ, Flanagan BM, Larroque O (2011). Impact of down-regulation of starch branching enzyme IIb in rice by artificial microRNA- and hairpin RNA-mediated RNA silencing. J Exp Bot.

[102] Kubo A, Akdogan G, Nakaya M, Shojo A, Suzuki S, Satoh H (2010). Structure, physical, and digestive properties of starch from wx/ae double-mutant rice. J Agric Food Chem.

[103] Tsuiki K, Fujisawa H, Itoh A, Sato M, Fujita N (2016). Alterations of starch structure lead to increased resistant starch of steamed rice: identification of high resistant starch rice lines. J Cereal Sci.

[104] Cummings JH, Stephen AM (2007). Carbohydrate terminology and classification. Eur J Clin Nutr.

[105] Yousefi AR, Razavi SM, Norouzy A (2015). In vitro gastrointestinal digestibility of native, hydroxypropylated and cross-linked wheat starches. Food Funct.

[106] Remya R, Jyothi AN, Sreekumar J (2017). Comparative study of RS4 type resistant starches derived from cassava and potato starches via octenyl succinylation. Starch.

[107] Hasjim J, Lee SO, Hendrich S, Setiawan S, Ai Y, Jane JL (2010). Characterization of a novel resistant-starch and its effects on postprandial plasma-glucose and insulin responses. Cereal Chem.

[108] Fuentes-Zaragoza E, Sánchez-Zapata E, Sendra E, Sayas E, Navarro C, Fernández-López J (2011). Resistant starch as prebiotic: a review. Starch.

[109] Ai Y, Hasjim J, Jane JL (2013). Effects of lipids on enzymatic hydrolysis and physical properties of starch. CarbohydrPolym.

[110] Zhang B, Dhital S, Gidley MJ (2015). Densely packed matrices as rate determining features in starch hydrolysis. Trends Food Sci Technol.

[111] Axelsen M, Arvidsson Lenner R, Lönnroth P, Smith U (1999). Breakfast glycaemic response in patients with type 2 diabetes: effects of bedtime dietary carbohydrates. Eur J Clin Nutr.

[112] Robertson MD, Bickerton AS, Dennis AL, Vidal H, Frayn KN (2005). Insulin-sensitizing effects of dietary resistant starch and effects on skeletal muscle and adipose tissue metabolism. Am J Clin Nutr.

[113] Mann JI, De Leeuw I, Hermansen K, Karamanos B, Karlström B, Katsilambros N (2004). Evidence-based nutritional approaches to the treatment and prevention of diabetes mellitus. NutrMetab Cardiovasc Dis.

[114] Mann J, Cummings JH, Englyst HN, Key T, Liu S, Riccardi G (2007). FAO/WHO scientific update on carbohydrates in human nutrition: conclusions. Eur J Clin Nutr.

[115] Brubaker PL, Ohayon EL, D’Alessandro LM, Norwich KH (2007). A mathematical model of the oral glucose tolerance test illustrating the effects of the incretins. Ann Biomed Eng.

[116] Holst JJ, Gribble F, Horowitz M, Rayner CK (2016). Roles of the gut in glucose homeostasis. Diabetes Care.

[117] Evert AB, Boucher JL, Cypress M, Dunbar SA, Franz MJ, Mayer-Davis EJ (2014). Nutrition therapy recommendations for the management of adults with diabetes. Diabetes Care.

[118] Takahashi K, Fujita H, Fujita N, Takahashi Y, Kato S, Shimizu T (2022). A pilot study to assess glucose, insulin, and incretin responses following novel high resistant starch rice ingestion in healthy men. Diabetes Ther.

[119] Maki KC, Pelkman CL, Finocchiaro ET, Kelley KM, Lawless AL, Schild AL (2012). Resistant starch from high-amylose maize increases insulin sensitivity in overweight and obese men. J Nutr.

[120] Pfister B, Zeeman SC (2016). Formation of starch in plant cells. Cell Mol Life Sci.

[121] Ang K, Bourgy C, Fenton H, Regina A, Newberry M, Diepeveen D (2020). Noodles made from high amylose wheat flour attenuate postprandial glycaemia in healthy adults. Nutrients.

[122] Roach PJ, Depaoli-Roach AA, Hurley TD, Tagliabracci VS (2012). Glycogen and its metabolism: some new developments and old themes. Biochem J.

[123] Seung D (2020). Amylose in starch: towards an understanding of biosynthesis, structure and function. New Phytol.

[124] Nielsen MM, Ruzanski C, Krucewicz K, Striebeck A, Cenci U, Ball SG (2018). Crystal structures of the catalytic domain of Arabidopsis thaliana starch synthase IV, of granule bound starch synthase from CLg1 and of granule bound starch synthase I of Cyanophoraparadoxa illustrate substrate recognition in starch synthases. Front Plant Sci.

[125] Cuesta-Seijo JA, Nielsen MM, Ruzanski C, Krucewicz K, Beeren SR, Rydhal MG (2015). In vitro biochemical characterization of all barley endosperm starch synthases. Front Plant Sci.

[126] Denyer K, Waite D, Motawia S, Møller BL, Smith AM (1999). Granule-bound starch synthase I in isolated starch granules elongates malto-oligosaccharides processively. Biochem J.

[127] Delvallé D, Dumez S, Wattebled F, Roldán I, Planchot V, Berbezy P (2005). Soluble starch synthase I: a major determinant for the synthesis of amylopectin in Arabidopsis thaliana leaves. Plant J.

[128] Fujita N, Yoshida M, Kondo T, Saito K, Utsumi Y, Tokunaga T (2007). Characterization of SSIIIa-deficient mutants of rice: the function of SSIIIa and pleiotropic effects by SSIIIa deficiency in the rice endosperm. Plant Physiol.

[129] Inouchi N, Glover DV, Fuwa H (1987). Chain length distribution of amylopectins of several single mutants and the normal counterpart, and sugary-1 phytoglycogen in maize (Zea mays L). Starch.

[130] Lloyd JR, Landschütze V, Kossmann J (1999). Simultaneous antisense inhibition of two starch-synthase isoforms in potato tubers leads to accumulation of grossly modified amylopectin. Biochem J.

[131] Pfister B, Lu KJ, Eicke S, Feil R, Lunn JE, Streb S (2014). Genetic evidence that chain length and branch point distributions are linked determinants of starch granule formation in Arabidopsis. Plant Physiol.

[132] Szydlowski N, Ragel P, Hennen-Bierwagen TA, Planchot V, Myers AM, Mérida A (2011). Integrated functions among multiple starch synthases determine both amylopectin chain length and branch linkage location in Arabidopsis leaf starch. J Exp Bot.

[133] Wang YJ, White PJ Pollak L, Jane JL (1993). Characterization of starch structures of 17 maize endosperm mutant genotypes with Oh43 inbred line background. Cereal Chem.

[134] Zhang X, Myers AM, James MG (2005). Mutations affecting starch synthase III in Arabidopsis alter leaf starch structure and increase the rate of starch synthesis. Plant Physiol.

[135] Zhang X, Szydlowski N, Delvallé D, D’Hulst C, James MG, Myers AM (2008). Overlapping functions of the starch synthases SSII and SSIII in amylopectin biosynthesis in Arabidopsis. BMC Plant Biol.

[136] Crumpton-Taylor M, Pike M, Lu KJ, Hylton CM, Feil R, Eicke S (2013). Starch synthase 4 is essential for coordination of starch granule formation with chloroplast division during Arabidopsis leaf expansion. New Phytol.

[137] Roldán I, Wattebled F, Mercedes Lucas M, Delvallé D, Planchot V, Jiménez S (2007). The phenotype of soluble starch synthase IV defective mutants of Arabidopsis thaliana suggests a novel function of elongation enzymes in the control of starch granule formation. Plant J.

[138] Abt MR, Pfister B, Sharma M, Eicke S, Bürgy L, Neale I (2020). STARCH SYNTHASE5, a noncanonical starch synthase-like protein, promotes starch granule initiation in Arabidopsis. Plant Cell.

[139] Burton RA, Bewley JD, Smith AM, Bhattacharyya MK, Tatge H, Ring S (1995). Starch branching enzymes belonging to distinct enzyme families are differentially expressed during pea embryo development. Plant J.

[140] Sawada T, Francisco PB Jr, Aihara S, Utsumi Y, Yoshida M, Oyama Y (2009). Chlorella starch branching enzyme II (BEII) can complement the function of BEIIb in rice endosperm. Plant Cell Physiol.

[141] Vos-Scheperkeuter GH, de Wit JG, Ponstein AS, Feenstra WJ, Witholt B (1989). Immunological comparison of the starch branching enzymes from potato tubers and maize kernels. Plant Physiol.

[142] Kossmann J, Visser RG, Müller-Röber B, Willmitzer L, Sonnewald U (1991). Cloning and expression analysis of a potato cDNA that encodes branching enzyme: evidence for co-expression of starch biosynthetic genes. Mol Gen Genet.

[143] Larsson CT, Hofvander P, Khoshnoodi J, Ek B, Rask L, Larsson H (1996). Three isoforms of starch synthase and two isoforms of branching enzyme are present in potato tuber starch. Plant Sci.

[144] Safford R, Jobling SA, Sidebottom CM, Westcott RJ, Cooke D, Tober KJ (1998). Consequences of antisense RNA inhibition of starch branching enzyme activity on properties of potato starch. CarbohydrPolym.

[145] Jobling SA, Schwall GP, Westcott RJ, Sidebottom CM, Debet M, Gidley MJ (1999). A minor form of starch branching enzyme in potato (Solanum tuberosum L) tubers has a major effect on starch structure: cloning and characterisation of multiple forms of SBE A. Plant J.

[146] Mizuno K, Kawasaki T, Shimada H, Satoh H, Kobayashi E, Okumura S (1993). Alteration of the structural properties of starch components by the lack of an isoform of starch branching enzyme in rice seeds. J Biol Chem.

[147] Guan H, Li P, Imparl-Radosevich J, Preiss J, Keeling P (1997). Comparing the properties of Escherichia coli branching enzyme and maize branching enzyme. Arch BiochemBiophys.

[148] Nozaki K, Hamada S, Nakamori T, Ito H, Sagisaka S, Yoshida H (2001). Major isoforms of starch branching enzymes in premature seeds of kidney bean (Phaseolus vulgaris L). BiosciBiotechnolBiochem.

[149] Rydberg U, Andersson L, Andersson R, Aman P, Larsson H (2001). Comparison of starch branching enzyme I and II from potato. Eur J Biochem.

[150] Nakamura Y, Utsumi Y, Sawada T, Aihara S, Utsumi C, Yoshida M (2010). Characterization of the reactions of starch branching enzymes from rice endosperm. Plant Cell Physiol.

[151] Hizukuri S (1986). Polymodal distribution of the chain lengths of amylopectins, and its significance. Carbohydr Res.

[152] Vamadevan V, Bertoft E, Seetharaman K (2013). On the importance of organization of glucan chains on thermal properties of starch. CarbohydrPolym.

[153] Burton RA, Zhang XQ, Hrmova M, Fincher GB (1999). A single limit dextrinase gene is expressed both in the developing endosperm and in germinated grains of barley. Plant Physiol.

[154] Delatte T, Umhang M, Trevisan M, Eicke S, Thorneycroft D, Smith SM (2006). Evidence for distinct mechanisms of starch granule breakdown in plants. J Biol Chem.

[155] Hussain H, Mant A, Seale R, Zeeman S, Hinchliffe E, Edwards A (2003). Three isoforms of isoamylase contribute different catalytic properties for the debranching of potato glucans. Plant Cell.

[156] Dinges JR, Colleoni C, James MG, Myers AM (2003). Mutational analysis of the pullulanase-type debranching enzyme of maize indicates multiple functions in starch metabolism. Plant Cell.

[157] Wattebled F, Dong Y, Dumez S, Delvallé D, Planchot V, Berbezy P (2005). Mutants of Arabidopsis lacking a chloroplastic isoamylase accumulate phytoglycogen and an abnormal form of amylopectin. Plant Physiol.

[158] Yun MS, Umemoto T, Kawagoe Y (2011). Rice debranching enzyme isoamylase3 facilitates starch metabolism and affects plastid morphogenesis. Plant Cell Physiol.

[159] Streb S, Delatte T, Umhang M, Eicke S, Schorderet M, Reinhardt D (2008). Starch granule biosynthesis in Arabidopsis is abolished by removal of all debranching enzymes but restored by the subsequent removal of an endoamylase. Plant Cell.

[160] Bhattacharyya MK, Smith AM, Ellis TH, Hedley C, Martin C (1990). The wrinkled-seed character of pea described by Mendel is caused by a transposon-like insertion in a gene encoding starch-branching enzyme. Cell.

[161] Satoh H, Omura T (1979). Induction of mutation by the treatment of fertilized egg cell with N-methyl-IV-nitrosourea in rice. J Fac Agric Kyushu Univ.

[162] Nishi A, Nakamura Y, Tanaka N, Satoh H (2001). Biochemical and genetic analysis of the effects of amylose-extender mutation in rice endosperm. Plant Physiol.

[163] Abe N, Asai H, Yago H, Oitome NF, Itoh R, Crofts N (2014). Relationships between starch synthase I and branching enzyme isozymes determined using double mutant rice lines. BMC Plant Biol.

[164] Kang HJ, Hwang IK, Kim KS, Choi HC (2003). Comparative structure and physicochemical properties of Ilpumbyeo, a high-quality japonica rice, and its mutant, Suweon 464. J Agric Food Chem.

[165] Zhu L, Gu M, Meng X, Cheung SC, Yu H, Huang J (2012). High-amylose rice improves indices of animal health in normal and diabetic rats. Plant Biotechnol J.

[166] Sun Y, Jiao G, Liu Z, Zhang X, Li J, Guo X (2017). Generation of high-amylose rice through CRISPR/Cas9-mediated targeted mutagenesis of starch branching enzymes. Front Plant Sci.

[167] Baysal C, Bortesi L, Zhu C, Farré G, Schillberg S, Christou P (2016). CRISPR/Cas9 activity in the rice OsBEIIb gene does not induce off-target effects in the closely related paralog OsBEIIa. Mol Breed.

[168] Baysal C, He W, Drapal M, Villorbina G, Medina V, Capell T (2020). Inactivation of rice starch branching enzyme IIb triggers broad and unexpected changes in metabolism by transcriptional reprogramming. Proc Natl Acad Sci U S A.

[169] Asai H, Abe N, Matsushima R, Crofts N, Oitome NF, Nakamura Y (2014). Deficiencies in both starch synthase IIIa and branching enzyme IIb lead to a significant increase in amylose in SSIIa-inactive japonica rice seeds. J Exp Bot.

[170] Vineyard ML, Bear RP (1952). Amylose content. Maize Genetic Cooperation Newsletter.

[171] Singletary GW, Banisadr R, Keeling PL (1997). Influence of gene dosage on carbohydrate synthesis and enzymatic activities in endosperm of starch-deficient mutants of maize. Plant Physiol.

[172] Arora A, Bhamare D, Das AK, Dixit S, Venadan S, Yathish KR (2024). Development of high-amylose maize (Zea mays L) genotypes adapted to Indian conditions through molecular breeding. Crop Pasture Sci.

[173] Zhao Y, Li N, Li B, Li Z, Xie G, Zhang J (2015). Reduced expression of starch branching enzyme IIa and IIb in maize endosperm by RNAi constructs greatly increases the amylose content in kernel with nearly normal morphology. Planta.

[174] Guan S, Wang P, Liu H, Liu G, Ma Y, Zhao L (2011). Production of high-amylose maize lines using RNA interference in sbe2a. Afr J Biotechnol.

[175] Regina A, Berbezy P, Kosar-Hashemi B, Li S, Cmiel M, Larroque O (2015). A genetic strategy generating wheat with very high amylose content. Plant Biotechnol J.

[176] Hazard B, Zhang X, Colasuonno P, Uauy C, Beckles DM, Dubcovsky J (2012). Induced mutations in the starch branching enzyme II (SBEII) genes increase amylose and resistant starch content in durum wheat. Crop Sci.

[177] Hogg AC, Gause K, Hofer P, Martin JM, Graybosch RA, Hansen LE (2013). Creation of a high-amylose durum wheat through mutagenesis of starch synthase II (SSIIa). J Cereal Sci.

[178] Fahy B, Gonzalez O, Savva GM, Ahn-Jarvis JH, Warren FJ, Dunn J (2022). Loss of starch synthase IIIa changes starch molecular structure and granule morphology in grains of hexaploid bread wheat. Sci Rep.

[179] Slade AJ, McGuire C, Loeffler D, Mullenberg J, Skinner W, Fazio G (2012). Development of high amylose wheat through TILLING. BMC Plant Biol.

[180] Sestili F, Palombieri S, Botticella E, Mantovani P, Bovina R, Lafiandra D (2015). TILLING mutants of durum wheat result in a high amylose phenotype and provide information on alternative splicing mechanisms. Plant Sci.

[181] Botticella E, Sestili F, Hernandez-Lopez A, Phillips A, Lafiandra D (2011). High resolution melting analysis for the detection of EMS induced mutations in wheat SBEIIa genes. BMC Plant Biol.

[182] Regina A, Bird A, Topping D, Bowden S, Freeman J, Barsby T (2006). High-amylose wheat generated by RNA interference improves indices of large-bowel health in rats. Proc Natl Acad Sci U S A.

[183] Sestili F, Janni M, Doherty A, Botticella E, D’Ovidio R, Masci S (2010). Increasing the amylose content of durum wheat through silencing of the SBEIIa genes. BMC Plant Biol.

[184] Morell MK, Kosar-Hashemi B, Cmiel M, Samuel MS, Chandler P, Rahman S (2003). Barley sex6 mutants lack starch synthase IIa activity and contain a starch with novel properties. Plant J.

[185] Li Z, Li D, Du X, Wang H, Larroque O, Jenkins CL (2011). The barley amo1 locus is tightly linked to the starch synthase IIIa gene and negatively regulates expression of granule-bound starch synthetic genes. J Exp Bot.

[186] Regina A, Kosar-Hashemi B, Ling S, Li Z, Rahman S, Morell M (2010). Control of starch branching in barley defined through differential RNAi suppression of starch branching enzyme IIa and IIb. J Exp Bot.

[187] Carciofi M, Blennow A, Jensen SL, Shaik SS, Henriksen A, Buléon A (2012). Concerted suppression of all starch branching enzyme genes in barley produces amylose-only starch granules. BMC Plant Biol.

[188] Zhou W, Zhao S, He S, Ma Q, Lu X, Hao X (2020). Production of very-high-amylose cassava by post-transcriptional silencing of branching enzyme genes. J Integr Plant Biol.

[189] Schwall GP, Safford R, Westcott RJ, Jeffcoat R, Tayal A, Shi YC (2000). Production of very-high-amylose potato starch by inhibition of SBE A and B. Nat Biotechnol.

[190] Hofvander P, Andersson M, Larsson CT, Larsson H (2004). Field performance and starch characteristics of high-amylose potatoes obtained by antisense gene targeting of two branching enzymes. Plant Biotechnol J.

[191] Andersson M, Melander M, Pojmark P, Larsson H, Bülow L, Hofvander P (2006). Targeted gene suppression by RNA interference: an efficient method for production of high-amylose potato lines. J Biotechnol.

[192] Tuncel A, Corbin KR, Ahn-Jarvis J, Harris S, Hawkins E, Smedley MA (2019). Cas9-mediated mutagenesis of potato starch-branching enzymes generates a range of tuber starch phenotypes. Plant Biotechnol J.

[193] Zhao X, Jayarathna S, Turesson H, Fält AS, Nestor G, González MN (2021). Amylose starch with no detectable branching developed through DNA-free CRISPR-Cas9 mediated mutagenesis of two starch branching enzymes in potato. Sci Rep.

[194] Oka H, Morishima H. Wild and cultivated rice. In: Futsuhara Y, Kikuchi F, Matsuo T, Yamaguchi H, eds. Science of the Rice Plant, Genetics. Tokyo: Nobunkyo; 1997.

[195] Nakamura Y, Sakurai A, Inaba Y, Kimura K, Iwasawa N, Nagamine T (2002). The fine structure of amylopectin in endosperm from Asian cultivated rice can be largely classified into two classes. Starch.

[196] Inouchi N, Hibiu H, Li T, Horibata T, Fuwa H, Itani T (2005). Structure and properties of endosperm starches from cultivated rice of Asia and other countries. J Appl Glycosci.

[197] Hirano HY, Sano Y (1998). Enhancement of Wx gene expression and the accumulation of amylose in response to cool temperatures during seed development in rice. Plant Cell Physiol.

[198] Wang ZY, Zheng FQ, Shen GZ, Gao JP, Snustad DP, Li MG (1995). The amylose content in rice endosperm is related to the post-transcriptional regulation of the waxy gene. Plant J.

[199] Isshiki M, Morino K, Nakajima M, Okagaki RJ, Wessler SR, Izawa T (1998). A naturally occurring functional allele of the rice waxy locus has a GT to TT mutation at the 5’ splice site of the first intron. Plant J.

[200] Sano Y (1984). Differential regulation of waxy gene expression in rice endosperm. Theor Appl Genet.

[201] Umemoto T, Yano M, Satoh H, Shomura A, Nakamura Y (2002). Mapping of a gene responsible for the difference in amylopectin structure between japonica-type and indica-type rice varieties. Theor Appl Genet.

[202] Nakamura Y, Francisco PB Jr, Hosaka Y, Sato A, Sawada T, Kubo A (2005). Essential amino acids of starch synthase IIa differentiate amylopectin structure and starch quality between japonica and indica rice varieties. Plant Mol Biol.

[203] Crofts N, Domon A, Miura S, Hosaka Y, Oitome NF, Itoh A (2022). Starch synthases SSIIa and GBSSI control starch structure but do not determine starch granule morphology in the absence of SSIIIa and SSIVb. Plant Mol Biol.

[204] Wang K, Wambugu PW, Zhang B, Wu AC, Henry RJ, Gilbert RG (2015). The biosynthesis, structure and gelatinization properties of starches from wild and cultivated African rice species (Oryza barthii and Oryza glaberrima). CarbohydrPolym.

[205] Wambugu P, Ndjiondjop MN, Furtado A, Henry R (2018). Sequencing of bulks of segregants allows dissection of genetic control of amylose content in rice. Plant Biotechnol J.

[206] Fujita N, Satoh R, Hayashi A, Kodama M, Itoh R, Aihara S (2011). Starch biosynthesis in rice endosperm requires the presence of either starch synthase I or IIIa. J Exp Bot.

[207] Merritt NR (1967). A new strain of barley with starch of high amylose content. J Inst Brew.

[208] Eslick RF, Ullrich SE (1977). A characterization of three high lysine mutants in relation to their normal isotypes and their environmental response. Barley Newsletter.

[209] Clarke B, Liang R, Morell MK, Bird AR, Jenkins CL, Li Z (2008). Gene expression in a starch synthase IIa mutant of barley: changes in the level of gene transcription and grain composition. FunctIntegr Genomics.

[210] Topping DL, Morell MK, King RA, Li Z, Bird AR, Noakes M (2003). Resistant starch and health—Himalaya 292, a novel barley cultivar to deliver benefits to consumers. Starch.

[211] Yamamori M, Fujita S, Hayakawa K, Matsuki J, Yasui T (2000). Genetic elimination of a starch granule protein, SGP-1, of wheat generates an altered starch with apparent high amylose. Theor Appl Genet.

[212] Shimbata T, Nakamura T, Vrinten P, Saito M, Yonemaru J, Seto Y (2005). Mutations in wheat starch synthase II genes and PCR-based selection of a SGP-1 null line. Theor Appl Genet.

[213] Konik-Rose C, Thistleton J, Chanvrier H, Tan I, Halley P, Gidley M (2007). Effects of starch synthase IIa gene dosage on grain, protein and starch in endosperm of wheat. Theor Appl Genet.

[214] Hylton C, Smith AM (1992). The rb mutation of peas causes structural and regulatory changes in ADP glucose pyrophosphorylase from developing embryos. Plant Physiol.

[215] Kossmann J, Abel GJ, Springer F, Lloyd JR, Willmitzer L (1999). Cloning and functional analysis of a cDNA encoding a starch synthase from potato (Solanum tuberosum L) that is predominantly expressed in leaf tissue. Planta.

[216] Craig J, Lloyd JR, Tomlinson K, Barber L, Edwards A, Wang TL (1998). Mutations in the gene encoding starch synthase II profoundly alter amylopectin structure in pea embryos. Plant Cell.

[217] Wang TL, Hadavizideh A, Harwood A, Welham TJ, Harwood WA, Faulks R (1990). An analysis of seed development in Pisum sativum XIII The chemical induction of storage product mutants. Plant Breed.

[218] Lamprecht H (1956). Pisum sativum L oder P arvense L eine nomenklatorische studie auf genetischer basis. Agriculture Horticulture Gen.

[219] White OE (1917). Studies of inheritance in Pisum II The present state of knowledge of heredity and variation in peas. Proc Am Philos Soc.

[220] Boyer CD, Preiss J (1978). Multiple forms of starch branching enzyme of maize: evidence for independent genetic control. BiochemBiophys Res Commun.

[221] Stinard PS, Robertson DS, Schnable PS (1993). Genetic isolation, cloning, and analysis of a mutator-induced, dominant antimorph of the maize amylose extender1 locus. Plant Cell.

[222] Satoh H, Nishi A, Yamashita K, Takemoto Y, Tanaka Y, Hosaka Y (2003). Starch-branching enzyme I-deficient mutation specifically affects the structure and properties of starch in rice endosperm. Plant Physiol.

[223] Regina A, Kosar-Hashemi B, Li Z, Pedler A, Mukai Y, Yamamoto M (2005). Starch branching enzyme IIb in wheat is expressed at low levels in the endosperm compared to other cereals and encoded at a non-syntenic locus. Planta.

[224] Yao Y, Thompson DB, Guiltinan MJ (2004). Maize starch-branching enzyme isoforms and amylopectin structure In the absence of starch-branching enzyme IIb, the further absence of starch-branching enzyme Ia leads to increased branching. Plant Physiol.

[225] Tetlow IJ, Emes MJ (2014). A review of starch-branching enzymes and their role in amylopectin biosynthesis. IUBMB Life.

[226] Wada T, Yamaguchi O, Miyazaki M, Miyahara K, Ishibashi M, Aihara T (2018). Development and characterization of a new rice cultivar, ‘Chikushi-kona 85’, derived from a starch-branching enzyme IIb-deficient mutant line. Breed Sci.

[227] Li M, Piao JH, Tian Y, Li WD, Li KJ, Yang XG (2010). Postprandial glycaemic and insulinaemic responses to GM-resistant starch-enriched rice and the production of fermentation-related H2 in healthy Chinese adults. Br J Nutr.

[228] Corrado M, Ahn-Jarvis JH, Fahy B, Savva GM, Edwards CH, Hazard BA (2022). Effect of high-amylose starch branching enzyme II wheat mutants on starch digestibility in bread, product quality, postprandial satiety and glycaemic response. Food Funct.

[229] Åkerberg A, Liljeberg H, Björck I (1998). Effects of amylose/amylopectin ratio and baking conditions on resistant starch formation and glycaemic indices. J Cereal Sci.

[230] Panlasigui LN, Thompson LU, Juliano BO, Perez CM, Yiu SH, Greenberg GR (1991). Rice varieties with similar amylose content differ in starch digestibility and glycemic response in humans. Am J Clin Nutr.

[231] Van Hung P, Yamamori M, Morita N (2005). Formation of enzyme-resistant starch in bread as affected by high-amylose wheat flour substitutions. Cereal Chem.

[232] De Arcangelis E, Angelicola M, Trivisonno MC, Iacovino S, Falasca L, Lafiandra D (2022). High-amylose bread wheat and its effects on cooking quality and nutritional properties of pasta. Int J Food Sci Technol.

[233] Nakamura S, Ohtsubo K (2010). Influence of physicochemical properties of rice flour on oil uptake of tempura frying batter. BiosciBiotechnolBiochem.

[234] Itoh Y, Crofts N, Abe M, N FO, Fujita N (2016). Screening method for novel rice starch mutant lines prepared by introducing gene encoding starch synthase IIa and granule-bound starch synthase I from indica cultivar into a branching enzyme IIb-deficient mutant line. J Appl Glycosci (1999).

[235] Itoh Y, Crofts N, Abe M, Hosaka Y, Fujita N (2017). Characterization of the endosperm starch and the pleiotropic effects of biosynthetic enzymes on their properties in novel mutant rice lines with high resistant starch and amylose content. Plant Sci.

[236] Lloyd JR, Kossmann J (2018). Starch trek: the search for yield. Front Plant Sci.

[237] Mwenje E, Chimwamurombe P. Cisgenesis: a promising alternative crop improvement technology for biodiversity, environment and ecosystem risks associated with transgenics. In: Chaurasia A, Kole C, eds. Cisgenic Crops: Safety, Legal and Social Issues. Cham: Springer; 2023. p. 31-42. doi: 10.1007/978-3-031-10721-4_2.

[238] Rock JS, Schnurr MA, Kingiri A, Ely A, Glover D, Stone GD (2023). The knowledge politics of genome editing in Africa. Elementa (Wash D C).

[239] Adegbaju MS, Ajose T, Adegbaju IE, Omosebi T, Ajenifujah-Solebo SO, Falana OY (2024). Genetic engineering and genome editing technologies as catalyst for Africa’s food security: the case of plant biotechnology in Nigeria. Front Genome Ed.

[240] Wolt JD, Wang K, Yang B (2016). The regulatory status of genome-edited crops. Plant Biotechnol J.

